# The Fluoride Anion-Catalyzed Sulfurization of Thioketones with Elemental Sulfur Leading to Sulfur-Rich Heterocycles: First Sulfurization of Thiochalcones †

**DOI:** 10.3390/molecules26040822

**Published:** 2021-02-05

**Authors:** Grzegorz Mlostoń, Jakub Wręczycki, Katarzyna Urbaniak, Dariusz M. Bieliński, Heinz Heimgartner

**Affiliations:** 1 Department of Organic and Applied Chemistry, Faculty of Chemistry, University of Lodz, 12 Tamka Street, 91-403 Lodz, Poland; katarzyna.urbaniak@chemia.uni.lodz.pl; 2 Institute of Polymer and Dye Technology, Faculty of Chemistry, Lodz University of Technology, 12/16 Stefanowskiego Street, 90-924 Lodz, Poland; dariusz.bielinski@p.lodz.pl; 3 Department of Chemistry, University of Zurich, Winterthurerstrasse 190, CH-8057 Zurich, Switzerland; heinz.heimgartner@chem.uzh.ch

**Keywords:** thioketones, thiochalcones, fluoride anion, elemental sulfur, sulfur heterocycles

## Abstract

Fluoride anion was demonstrated as a superior activator of elemental sulfur (S_8_) to perform sulfurization of thioketones leading to diverse sulfur-rich heterocycles. Due to solubility problems, reactions must be carried out either in THF using tetrabutylammonium fluoride (TBAF) or in DMF using cesium fluoride (CsF), respectively. The reactive sulfurizing reagents are in situ generated, nucleophilic fluoropolysulfide anions FS_(8−x)_^−^, which react with the C=S bond according to the carbophilic addition mode. Dithiiranes formed thereby, existing in an equilibrium with the ring-opened form (diradicals/zwitterions) are key-intermediates, which undergo either a step-wise dimerization to afford 1,2,4,5-tetrathianes or an intramolecular insertion, leading in the case of thioxo derivatives of 2,2,4,4-tetramethylcyclobutane-1,3-dione to ring enlarged products. In reactions catalyzed by TBAF, water bounded to fluoride anion via H-bridges and forming thereby its stable hydrates is involved in secondary reactions leading, e.g., in the case of 2,2,4,4-tetramethyl-3-thioxocyclobutanone to the formation of some unexpected products such as the ring enlarged dithiolactone and ring-opened dithiocarboxylate. In contrast to thioketones, the fluoride anion catalyzed sulfurization of their α,β-unsaturated analogues, i.e., thiochalcones is slow and inefficient. However, an alternative protocol with triphenylphosphine (PPh_3_) applied as a catalyst, offers an attractive approach to the synthesis of 3*H*-1,2-dithioles via 1,5-dipolar electrocyclization of the in situ-generated α*,*β-unsaturated thiocabonyl *S*-sulfides. All reactions occur under mild conditions and can be considered as attractive methods for the preparation of sulfur rich heterocycles with diverse ring-size.

## 1. Introduction

It is a well-known fact that large amounts of elemental sulfur (S_8_) are available as a side product of diverse technological processes, and its further usage in a sustainable cycle of conversions leading to useful, organic compounds represents an important and challenging problem for chemical industry and related branches [1]. Sulfurization reactions performed with elemental sulfur and leading to cyclic or acyclic organic compounds constitute an interesting and practically relevant topic in the current organic chemistry of sulfur. Results, which were mainly published in the recent two decades, are summarized and compared in an excellent, comprehensive review [2].

**Figure 1:**
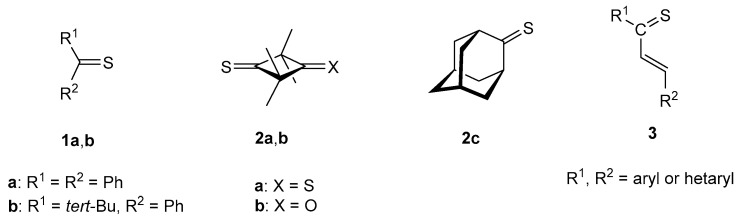
Aromatic and cycloaliphatic thioketones **1** and **2**, and thiochalcones **3** applied in the study.

Reactions of thiocarbonyl compounds with elemental sulfur and their conversion into sulfur-rich heterocycles attract attention since many decades. Aromatic, aliphatic/aromatic or cycloaliphatic thioketones **1**, and **2**, e.g., thiobenzophenone (**1a**) and (*tert*-butyl) phenyl thioketone (**1b**), as well as cycloaliphatic analogues such as 2,2,4,4-tetramethylcyclobutane-1,3-dithione (**2a**), 2,2,4,4-tetramethyl-3-thioxocyclobutanone (**2b**) or adamantanethione (**2c**) are known to undergo sulfurization in the presence of a nucleophilic catalyst or upon heating without a catalyst in an appropriate organic solvent [3,4,5,6,7,8] (Figure 1). On the other hand, sulfurization of thiochalcones **3** (α*,*β-unsaturated thioketones) as substrates for the preparation of sulfur-rich heterocycles has not yet been reported.

**Figure 2:**
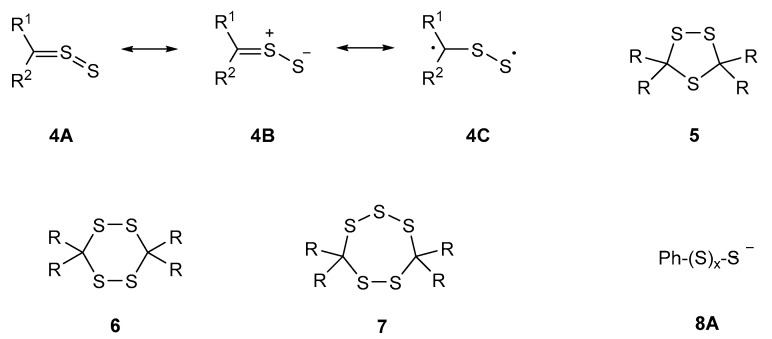
Thiocarbonyl *S*-sulfides **4**, the sulfur-rich heterocycles 1,2,4-trithiolanes **5**, 1,2,4,5-tetrathianes **6**, and 1,2,3,5,6-pentathiepanes **7**, as well as phenylpolysulfide anions **8A**.

In many cases, the mechanistic interpretation of the observed reactions course was based on the assumption that the transient thiocarbonyl *S*-sulfides (thiosulfines) **4** [9], generated via the transfer of a sulfur atom to the C=S bond, and considered as ‘sulfur-rich’ representatives of so called ‘sulfur-centered 1,3-dipoles’, play the crucial role in the formation of sulfur heterocycles, such as the corresponding 1,2,4-trithiolanes **5** [10] (Figure 2). On the other hand, the larger rings, e.g., 1,2,4,5-tetrathianes **6** or 1,2,3,5,6-pentathiepanes **7**, are formed via more complex processes, but the reaction mechanisms are not fully rationalized to date and require further studies [3,4,11].

For example, in some instances, sulfurization of thioketones performed in the presence of a nucleophilic catalyst, e.g., sodium or potassium thiophenolate, were explained via involvement of in situ-generated, reactive polysulfide anions of type **8A** [3,4]. Thus, Huisgen and Rapp reported that elemental sulfur reacts with thiobenzophenone (**1a**) in boiling acetone, in the presence of catalytic amounts of sodium thiophenolate, yielding 3,3,6,6-tetraphenyl-1,2,4,5-tetrathiane (**6a**) (R = Ph) as the exclusive product [3]. The same authors demonstrated that 3-thioxo-2,2,4,4-tetramethyl-cyclobutanone (**2b**) [4] and adamantanethione (**2c**) [3] also undergo sulfurization under the same conditions. However, the isolated products are either the bis-spirocyclic 1,2,4,5-tetrathianes **6b**/**6c** (R,R = **6b**: *spiro*-2,2,4,4-tetramethyl-3-oxocyclobutyl; **6c**: *spiro*-2-adamantyl) or 1,2,3,5,6-pentathiepanes **7a**/**7b** (R,R = **7a**: *spiro*-2,2,4,4-tetramethyl-3-oxocyclobutyl; **7b**: *spiro*-2-adamantyl), respectively.

Okuma also studied reactions of **2c** with elemental sulfur in the presence of catalytic amounts of triphenylphosphine sulfide (Ph_3_P=S) in boiling CHCl_3_ and isolated 1,2,4-trithiolane **5c** (R,R = *spiro*-2-adamantyl) in 59% yield [5]. In the same publication, sulfurization of **1a** with elemental sulfur in boiling xylene, in the presence of maleic anhydride as a trapping reagent for the postulated, intermediate thiobenzophenone *S*-sulfide (**4a**) (R_1_ = R_2_ = Ph), was also described, and the expected [3+2]-cycloadduct, i.e., 5,5-diphenyl-1,2-dithiolane-3,4-dicarboxylic acid anhydride, was obtained in 74% yield [5].

**Scheme 1:**
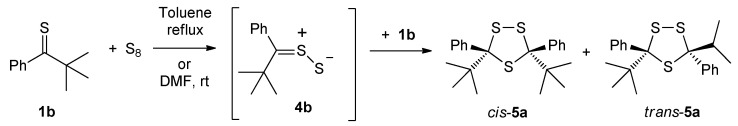
Reaction of *tert*-butyl phenyl thioketone (**1b**) with elemental sulfur S_8_ in boiling toluene or in DMF at rt (ref. [6,7]).

Nakayama et al. performed sulfurization of the cycloaliphatic thioketone **1b** with elemental sulfur without a nucleophilic catalyst using DMF or toluene as a solvent. Depending on the solvent, either a mixture of the isomeric 1,2,4-trithiolanes *cis*- and *trans*-**5a** (boiling toluene) or *cis*-**5a** as the sole product (DMF, rt), were obtained [6,7]. In these reactions, a plausible mechanistic explanation comprised the in situ-formation of thiocarbonyl *S*-sulfide **4b** and its subsequent stereoselective [3+2]-cycloaddition onto the unconverted thioketone **1b** (Scheme 1).

A similar reaction performed with *tert*-butyl 4-methylphenyl ketone in boiling pyridine and using tetraphosphorus decasulfide (P_4_S_10_) as a thionating and sulfurizing reagent, was described by Okuma et al. [8].

**Scheme 2:**
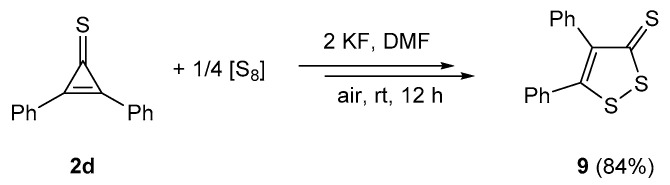
Fluoride anion-catalyzed sulfurization of diphenylcyclopropenethione (**2d**) (ref. [16]).

In spite of the fact that the fluoride anion is well known as a useful reagent acting not only as a unique catalyst for nucleophilic trifluoromethylation reactions [12], as a desilylating agent [13], but also as a strong base [14], its exploration as an activating reagent for elemental sulfur in the sulfur transfer reactions is limited to two reports only, which are known to date [15,16]. In one of them, Petrov and Marshall demonstrated the utility of the fluoride anion as a potent activator of elemental sulfur used for in situ-generation of reactive hexafluorothioacetone *S*-sulfide, which subsequently was trapped by ethylenic dipolarophiles yielding corresponding bis-trifluoromethylated 1,2-dithioles in high yields [15]. In the second publication, sulfurization of cyclopropenethione **2d** and its oxo-analogues with elemental sulfur in DMF solution at room temperature was described [16] (Scheme 2). In this study, potassium fluoride was used in two-fold excess as activator and the molar ratio of reagents **2d**/[S]/KF was optimized to 1:2:2. Under these conditions, 3-thioxo-1,2-dithiole **9** was obtained as the product of a cascade reaction and isolated in good yield (84%). A series of analogous Se-heterocycles was also obtained using elemental selenium instead of sulfur [16].

**Scheme 3:**
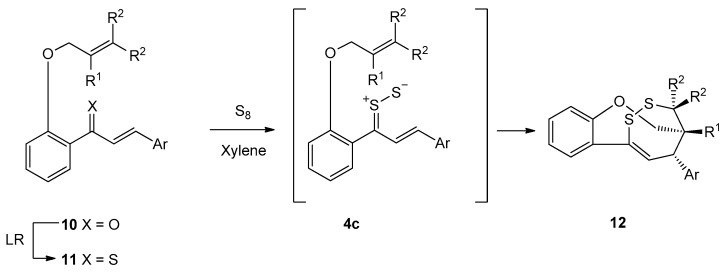
Reaction of thiochalcones **11** with sulfur yielding 1,2-dithiepanes **12** via intramolecular [5+2]-cycloaddition of intermediate thiocarbonyl *S*-sulfides **4c** (ref. [17]).

Unlike thioketones, similar sulfurization of α*,*β-unsaturated thioketones **3** (thiochalkones) with S_8_ are almost unknown. In an earlier publication, however, sulfurization of rather complex thiochalcones **11**, prepared in situ from corresponding ketones **10**, was carried out in non-polar xylene by treatment with S_8_. These reactions led to the polycyclic products **12**, which were formed as minor products, exclusively [17] (Scheme 3). The formation of the latter heterocycles was explained via a multi-step mechanism with the respective α,β-unsaturated thiocarbonyl *S*-sulfides **4c** as the reactive intermediates in the postulated intramolecular [5+2]-cycloaddition reactions.

The main goal of the present study was examination of the utility of the fluoride anion as a new type of a catalyst for sulfurization of thiobenzophenone (**1a**) and cycloaliphatic thioketones **2a**–**2c**. In extension of the study with thioketones, first sulfurization reactions of selected thiochalcones **3a**–**3d** were carried out by using elemental sulfur (S_8_) in the presence of a nucleophilic catalyst. Elucidation of the mechanisms of the studied reactions, leading to the formation of sulfur-rich heterocycles with diverse ring size, was of primary interest.

## 2. Results and Discussion

### 2.1. Conversions of Thioketones **2a** and **2b** upon Tretament with Catalytic Amounts of TBAF in the Absence of Elmental Sulfur

**Scheme 4:**
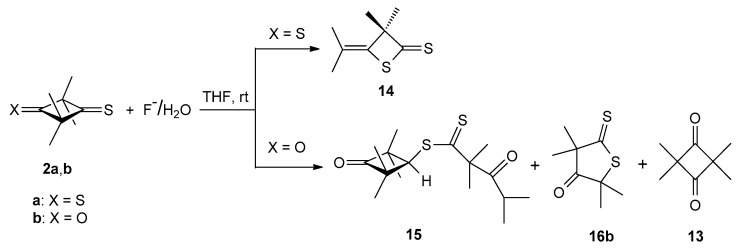
Comparison of unusual conversions of thioketones **2a** and **2b** upon treatment with catalytic amounts of TBAF in THF solution at rt in the absence of S_8_.

In an earlier publication, unexpected conversions of thiobenzophenone (**1a**) and cycloaliphatic thioketones **2a**–**2c** upon treatment with catalytic amounts of TBAF in THF solution were reported [18]. The obtained results demonstrated that the type of products formed in each reaction strongly depended on the type of thioketone used in the studied experiment. Interestingly enough, two structurally similar cycloaliphatic thioketones **2a** and **2b**—derived from 2,2,4,4-tetramethylcyclobutane-1,3-dione (**13**)—gave completely different sets of products. Whereas in the case of dithione **2a** the only product was the isomeric thiolactone **14**, the monothione **2b** afforded a mixture of the unusual dithiocarboxylate **15** and the parent dione **13** (Scheme 4).

Due to the importance of this observation, both experiments were repeated in the initial stages of the present study. The already described isomerization of **2a** leading to **14** as the sole product was fully confirmed. However, in the experiment with **2b** we found that along with **13** and **15** also the ring-enlarged five-membered dithiolactone **16b** was present in the crude mixture. Moreover, the molar ratio of dione **13** and dithiolactone **16b** was ca. 1:1 and this finding suggests that the monothione **2b** can be an actual source of sulfur incorporated into the four-membered ring of another molecule of the same substrate.

The mechanism of the isomerization of **2a** induced by fluoride anion presented in [18] is a plausible explanation of this conversion. On the other side, the formation of dithioester **15**, which requires reduction of the C=S bond of **2b**, as well as the intermolecular transfer of the sulfur atom from one molecule of **2b** to another one in the formation of **16b**, has not convincingly been rationalized yet. Searching for a correct explanation, an additional experiment with attempted conversion of **2b** using catalytic amounts of flame-dried CsF in dry DMF has also been performed. To our surprise, no effect was observed even after ca. 2 h at rt. This situation changed immediately after addition of a drop of water to the solution, which rapidly changed the color to orange indicating thereby conversion of **2b**. Subsequent control of the composition of products by means of ^1^H-NMR spectroscopy confirmed the formation of the mixture of three known products, namely **13**, **15**, and **16b.** This result clearly demonstrated that the unexpected conversion of **2b** can be performed only in the presence of water and the involvement of the hydrated fluoride anion in the reaction solution plays a crucial role in the formation of **15** and **16b**. Notably, TBAF is known to exist as a hydrated complex and the complete removal of water requires a special procedure [19].

### 2.2. Activation of Elemental Sulfur with Fluoride Anion

**Scheme 5:**
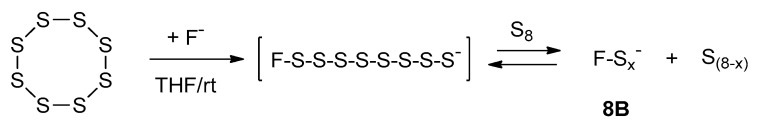
Ring cleavage of the S_8_ crown and formation of a mixture of fluoropolysulfide anions **8B**.

It is very likely that the activation of elemental sulfur with fluoride anion comprises cleavage of the S_8_-ring leading to a mixture of red-colored, highly nucleophilic fluoropolysulfide anions **8B** with variable length of the sulfur chain S_x_ (Scheme 5).

It is worth of mentioning that in the case of TBAF complete removal of water forming ‘fluoride anion hydrates’ is a difficult problem and some amounts of water are still present in the solution [20]. In contrast, cesium fluoride, which can be used alternatively as a source of the fluoride anion, can be prepared as a water-free salt. However, due to solubility problems reactions with CsF have to be performed in dry DMF solution. In the sulfurization reaction of a thioketone, the C=S bond is believed to react with anions **8B** according to the ‘carbophilic’ mode of the initial nucleophilic attack.

### 2.3. Sulfurization of Thioketones with Elemental Sulfur

All experiments were carried out using optimized protocols elaborated in a series of preliminary test reactions. Magnetically stirred suspension of elemental sulfur in THF at rt dissolved immediately after addition of catalytic amounts of TBAF, and the obtained solution was colored red or olive-green, thereby indicating formation of fluoropolysufide anions **8B** with variable length of the S_x_ chain. Addition of a thioketone led to the change of the color within a few minutes, and the completion of the reaction was established by TLC (see Table 1 and Table 2). After pre-purification by column chromatography aimed at separation of the catalyst, the crude mixtures were analyzed in the ^1^H-NMR spectra with weighted standard (1,2-dichloroethane; Method A), and pure products were isolated on preparative plates (Method B). Alternatively, selected reactions were performed in DMF solution using pre-dried CsF as a catalyst.

#### 2.3.1. Sulfurization of ‘Dithione’ **2a** and ‘Monothione’ **2b**

**Figure 3:**
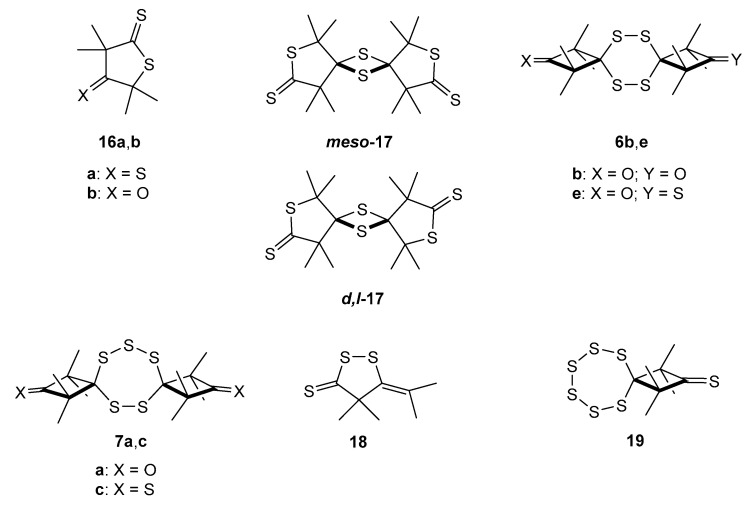
Products of sulfurization of thioketones **2a** and **2b** with elemental sulfur in the presence of TBAF in THF solution.

A series of experiments aimed at determination of the impact of the molar ratio of **2a** to sulfur on the structure of the formed products, was performed starting with 1:0.5, 1:1, 1:2, 1:4 or 1:8 (Entries A–E) mixtures of the two reagents. In the obtained crude mixtures both, known and hitherto unknown sulfur-rich heterocycles were initially identified by ^1^H-NMR spectroscopy (see Supplementary Materials part) and subsequently isolated as pure compounds (Figure 3).

In the first experiment, the reaction with a 1:0.5 molar ratio of **2a** and atomic [S] (Entry A) led to a mixture of the isomeric thiolactone **14** (63% yield) and the ring enlarged five-membered thiolactone **16a** (11%) (Figure 3, Table 1).

Enhancement of the sulfur amount in the 1:1 experiment (Entry B) resulted in a drastic change of composition of the products in the reaction mixture. The main component isolated as the most polar fraction showed in the ^1^H-NMR spectrum eight signals with approximately the same intensities located at 1.32, 1.35, 1.51, 1.51, 1.69, 1.71, 1.74, and 1.75 ppm. More important information was found in the ^13^C-NMR spectrum, which revealed the presence of two isomeric compounds with very similar patterns of absorptions. The most characteristic ones were those found at 248.18 and 248.39 ppm, which were attributed to two C=S groups of the dithiocarboxylate type. In addition, two signals at 78.54 and 78.71 ppm corresponded to non-equivalent C_sp3_ atoms connected with two sulfur atoms. In analogy to the ^1^H-NMR, eight signals between 26.32 and 30.66 ppm suggested the presence of structurally similar 16 Me groups. The elemental analysis confirmed the molecular formula C_16_H_24_S_6_, which can be attributed to a 1:1-mixture of the isomeric bis-spiro-1,3-dithietane derivatives *meso*- and *d,l*-**17**. All attempts to separate this mixture to obtain pure diastereoisomers, either by chromatography or fractional crystallization, were unsuccessful.

The less polar fraction formed a yellowish oil, which in the ^1^H-NMR spectrum showed the presence of only two broadened singlets located at 1.42 and 1.69 ppm. More information delivered the ^13^C-NMR spectrum with two low field absorptions at 243.0 and 216.6 ppm. Whereas the first signal could be attributed to the C=S group incorporated in the four-membered ring of cyclobutane, the second one was identified as the C=O unit of a cyclobutane moiety. In addition, two closely located signals with low intensity, found at 79.86 and 79.91 ppm, respectively, revealed the presence of two C_sp3_-atoms attached to two S-atoms like in the molecule of a 1,2,4,5-tetrathiane. As a matter of fact, elemental analysis confirmed the molecular formula C_16_H_24_OS_5_, which corresponds to the structure of the ‘non-symmetric’ dispiro-1,2,4,5-tetrathiane **6e** (Figure 3, Table 1). Apparently, the initially formed ‘symmetric’ analogue **6d** (not isolated) underwent hydrolysis in the reaction solution and one C=S group was converted into the C=O moiety.

Finally, the smallest and least polar fraction isolated after chromatography was identified as the known [4], five-membered dithiolactone **16a** (8%) (Table 1).

Increase of the molar excess of sulfur to 4 mol-equiv. (Entry D) changed dramatically the composition of the product mixture, and in this case the major fraction consisted of an orange oil, which in the ^1^H-NMR spectrum showed a complicated pattern of signals located in the 1.30–2.00 ppm region. In the spectrum registered at 90 °C in CHCl_2_–CHCl_2_ solution, four broadened signals emerged at 1.92, 1.98. 2.08, and 2.20 ppm, respectively, indicating thereby the existence of dynamic phenomena, like in the case of pentathiepane **7a** described by Huisgen et al. [4]. Elemental analysis confirmed the molecular formula C_16_H_24_S_7_ expected for the new pentathiepane **7c** and allowed to determine the yield of the isolated product to 46%. In another experiment carried out with **2a** and [S] in a ratio 1:2 (Entry C), pentathiepane **7c** was isolated in a lower yield (23%) and the already described sulfur heterocycles **14** (9%), **16a** (8%), and **18** (5%) were formed as side products.

Two more sulfur-rich heterocycles were separated as minor products. One of them was identified as the known five-membered dithiolactone **18** [21], and the structure of the second one was elucidated based on spectroscopic data and elemental analysis as hitherto unknown spirocyclic 1,2,3,4,5,6-hexathiepane **19** being a thioxo analogue of the corresponding oxo derivative, which was described in our earlier work [22]. Both compounds revealed very similar chemical shifts for the *spiro*-*C*-atoms: 95.6 (for **19**) and 92.2 (for the oxo analogue [22]) ppm, respectively. 

Notably, the same pentathiepane **7c** was isolated chromatographically in 54% yield as the sole product when the sulfurization of **2a** was carried out in DMF solution using dried CsF as a catalyst (Entry C′). In that experiment the molar ratio of **2a** and [S] was calculated to 1:2.

In contrast to the hitherto unexplored dithione **2a**, its monothione analogue **2b** was studied extensively by Huisgen and Rapp in sulfurization reactions with elemental sulfur using potassium thiophenolate as a catalyst [4]. The main goal of that study was the elucidation of the mechanism of the dimerization of in situ-generated intermediates leading to 1,2,4,5-tetrathiane **6b**. In our hands, reactions of **2b** with variable amounts of sulfur and performed with TBAF as a catalyst led virtually to similar products. Thus, in experiments starting with **2b** and [S] in a molar ratio 1:0.5 (Entry A) or 1:1 (Entry B), the only sulfur-rich compound identified in the crude mixture of products was **6b** accompanied by the above reported 3-oxopentanedithioate **15** and dithiolactone **16b**. In the 1:1 experiment, the yield of tetrathiane **6b** was higher (40%) (Table 1). Further enhancement of the amounts of sulfur to 1:2 (Entry C), 1:4 (Entry D), and finally 1:8 (Entry E) molar ratio suppressed completely the formation of other products and nothing but 1,2,3,5,6-pentathiepane **7a** was isolated in acceptable yields of 34%, 52%, and 60%, respectively. In a test experiment using pre-dried CsF (instead of TBAF) in DMF solution and starting with a 1:2 ratio of **2b** and atomic [S], the latter heterocycle was isolated in 43% yield (Entry C′). Spectroscopic and physico-chemical data of **7a** fitted well with those reported earlier [4]. In addition, the crystal structure of this compound showing a complex conformation of this seven-membered, sulfur-rich hetererocycle has also been reported in another publication [22]. In the present study, the structure of **7a** was redetermined by X-ray analysis and the crystallographic characterization of this sulfur-rich heterocycle could be refined thereby.

#### 2.3.2. Formation of Sulfur-Rich Heterocycles from Thioketones **2a** and **2b**; Mechanistic Interpretations

**Scheme 6:**
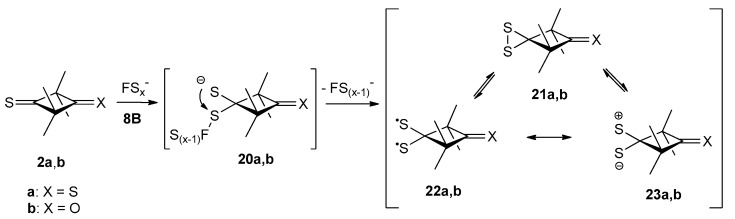
Initial, nucleophilic attack of fluoropolysulfide anion **8B** onto the C=S bond and subsequent ring closure leading to dithiiranes **21** as elusive intermediates.

It has to be underlined that in both series of experiments performed either with **2a** or **2b** and starting with a 1:0.5 molar ratio of a thioketone **2** and elemental sulfur [S], no traces at all of the corresponding 1,2,4-trithiolane of type **5** could be detected in the mixtures of products. These findings allow to exclude participation of thiocarbonyl *S*-sulfides (thiosulfines) **4A**–**C** derived from the studied thioketones **2** as reactive intermediates in the herein presented sulfurizations. Instead, these observations suggest to consider dithiiranes **21** and their ring opened forms, i.e., diradicals **22** (or zwitterions **23**), as elusive species involved in the formation of the isolated sulfur-rich heterocycles (Scheme 6). The parent dithiirane and its isomerization to dithioformic acid can serve as a model system helpful in the formulation of experimentally well founded rational description of the complex conversions observed in the present study [23].

It seems a plausible explanation that in situ-generated fluoropolysulfide anions **8B** undergo the carbophilic addition to the C=S bond forming the intermediate anion **20**. The latter extrudes a new fluoropolysulfide anion with a shortened sulfide chain yielding the elusive dithirane **21**, which may exist in an equilibrium with ring opened reactive species **22** and/or **23** (Scheme 6). 

**Scheme 7:**
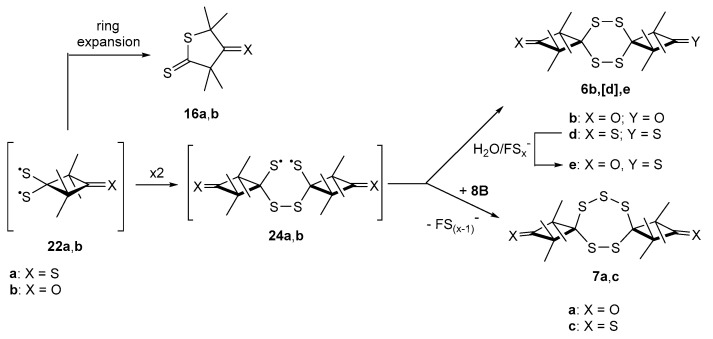
Step-wise formation of 1,2,4,5-tetrathianes **6** and 1,2,3,5,6-pentathiepanes **7**.

In the reaction performed with equimolar amounts of thioketone and sulfur, step-wise dimerization of the latter species leads to the formation of the 1,2,4,5-tetrathiane **6**. Alternatively, the intermediate species **22** undergo ring-expansion via intramolecular insertion of the S-atom into a C–C bond to form dithiolactones **16**. However, if there is a source of sulfur and fluoropolysulfide **8B** exists in the system, it can transfer one more sulfur atom to the intermediate **24** yielding finally 1,2,3,5,6-pentathiepanes **7** (Scheme 7). 

This interpretation seems to be more likely than an alternative pathway via a secondary ring opening/ring closure reaction sequence of the initially formed 1,2,4,5-tetrathiane derivative, after reaction with a polysulfide anion, leading either to 1,2,4-trithiolanes or 1,2,3,5,6-pentathiepanes. This pathway has been suggested in an earlier publication, dealing with similar problems of sulfurization reactions of some aromatic aldehydes [11].

**Scheme 8:**
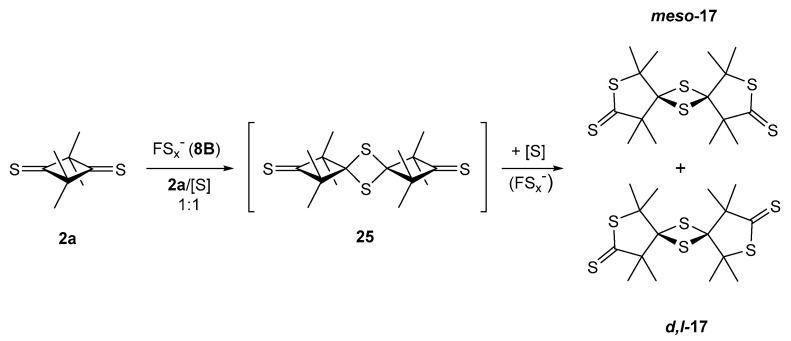
Postulated mechanism of the formation of diastereoisomeric bis-spiro-1,3-dithianes **17** via initial dimerization of the starting thioketone **2a**.

The unexpected formation of diastereoisomeric dispiro-1,3-dithietane **17** deserves also a comment. It seems likely that in analogy to the earlier observed BTAF/TMSCF_3_ induced dimerization of dithione **2a** [18] affording 1,3-dithietane **25**, also fluoropolysulfide anions **8B** can catalyze an analogous reaction. Now, each of the C=S bonds can react with **8B** and via addition/ring expansion sequence is converted in mixtures of isomeric, spiro-heterocycles **17** (Scheme 8). Alternatively, dimerization of the initially formed dithiolactone **16a**, induced by fluoride anion can be postulated. Notably, no formation of corresponding products of type **17**, starting with monothione **2b** was observed and this is one more feature, which differs behavior of both cycloaliphatic thioketones in sulfurization reactions.

**Scheme 9:**
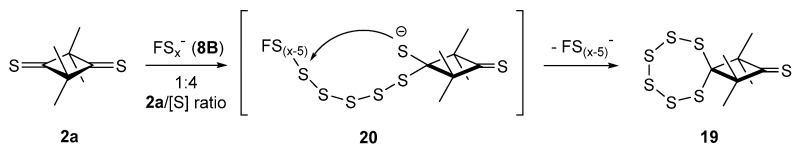
Mechanistic explanation of the formation of 1,2,3,4,5,6-hexathiepane **19**.

An alternative cyclization pathway of the initial anionic adduct **20**, formed via attack of anions **8B** onto the C=S group, leading to the hexathiepane **19**, is presented in Scheme 9. It demonstrates the known tendency for the formation of seven-membered rings consisting of sulfur and carbon atoms [22].

#### 2.3.3. Sulfurization of ‘Adamantanethione (**2c**) and Thiobenzophenone (**1a**)

**Figure 4:**
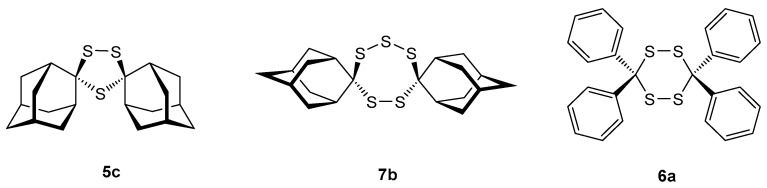
Three types of sulfur-rich heterocycles obtained by sulfurization of adamantanethione (**2c**) and thiobenzophenone (**1a**) with sulfur, catalyzed with fluoride anion.

In extension of the study performed with cycloaliphatic thioketones **2a** and **2b**, derived from the sterically crowded and ring congested 2,2,4,4-tetramethylcyclobutane-1,3-dione (**13**), sulfurization reactions of easily available thioketones, such as the cycloaliphatic adamantanethione (**2c**) and the aromatic thiobenzophenone (**1a**), were also tested in the fluoride anion catalyzed sulfurization reactions. In both cases, the reactions were carried out with TBAF as a catalyst in THF at rt, and required remarkably longer times for completion (see Table 2, Figure 4). Analysis of the crude mixtures of products by ^1^H-NMR spectroscopy and separation by preparative thin-layer chromatography led to the identification and isolation of three types of sulfur-rich heterocycles. Thus, in the case of **2c**, irrespective of the molar ratio of thioketone and sulfur, 1:1 (Entry B) or 1:2 (Entry C), only the symmetric 1,2,3,5,6-pentathiepane **7b** was obtained in moderate yields (37–51%). However, in the experiment with a 1:0.5 molar ratio (**2c**: [S], Entry A), 1,2,4-trithiolane **5c** was found in the mixture of products as the major component (59%). The same product was formed in DMF solution at rt exclusively, when the reaction was performed with CsF (Entry C′). Its presence suggests the appearance of adamantane-2-thione *S*-sulfide and subsequent trapping by another molecule of **2c** according to the [3+2]-cycloaddition mode as suggested in our earlier study [24]. Limited amounts of elemental sulfur available in the first experiment (Entry A) can suggest that the reaction leading to **5c** follows a hitherto unknown mechanism of the reaction of fluoride anion with **2c** mentioned in our earlier publication [18]. Nevertheless, formation of **7b** can results from the mechanism initiated by the attack of fluoropolysulfide anion onto the C=S bond according to the pathways presented in Scheme 7. Notably, no symmetric 1,2,4,5-tetrathiane derived from **2c** and obtained by Huisgen and Rapp [3] via sulfurization of the latter in reactions catalyzed with potassium thiophenolate, was observed in our experiments.

The fluoride anion catalyzed sulfurization of thiobenzophenone (**1a**) was performed starting with 1:1 or 1:2 molar ratio of **1a** to [S] (Entry B or Entry C, respectively). In both cases colorless products precipitated from THF solution, and the analysis of the obtained crude mixtures demonstrated that the literature known, symmetric 3,3,6,6-tetraphenyl-1,2,4,5-tetrathiane (**6a**) [3] was the sole product in both reactions, isolated in 28% and 52% yield, respectively. Remarkably, no ring expansion to the corresponding 1,2,3,5,6-pentathiepane in the experiment with molar excess of sulfur (1:2; Entry C) was observed. This observation suggests that this could be the effect of the limited solubility of **6a**, which precipitated from the THF solution in the course of the reaction. Notably, Huisgen and Rapp did not observe formation of this 1,2,3,5,6-pentathiepane either [3]. In the present study, along with **1a**, other well-known aromatic thioketones such as xanthione, thioxanthione and dibenzosuberenethione, which have been frequently applied in our studies, were tested in the fluoride anion catalyzed sulfurization, but in all cases no reaction was observed at rt, even after longer reaction time (up to one week). Particularly, thiofluorenone (9*H*-fluorene-9-thione) converted rapidly into the dimeric fluorenyliden-9-ene (tetrabenzo[5.5]fulvalene) in analogy to the result described in [18]. Thus, thiobenzophenone (**1a**) should be considered as an exceptional case showing superior tendency to undergo the reaction with fluoropolysulfide anion **8B** with subsequent ‘head-to-head’ dimerization of a reactive intermediate leading to tetrasubstituted 1,2,4,5-tetrathiane **6a**.

In accordance with the proposal presented in Scheme 9, a plausible mechanistic interpretation of this multi-step reaction is the initial formation of the unstable 3,3-diphenyl dithiirane (**21c**). The latter, after spontaneous cleavage of the S–S bond, converts into a reactive intermediate of type **24**, which undergoes step-wise dimerization leading to the 1,2,4,5-tetrathiane ring (Scheme 7).

### 2.4. Sulfurization of Thiochalcones **3**

In contrast to thioketones **1**/**2**, the α*,*β-unsaturated analogues, i.e., diaryl thiochalcones **3**, are much less investigated as potentially useful reagents in the cycloaddition chemistry. In fact, thiochalcones exhibit a far more complex nature in comparison with thioketones, which comprises their tendency to form two dimeric molecules existing in an equilibrium with the monomeric ones. The equilibrium concentration of monomeric and dimeric forms depends on the solvent polarity that makes thiochalcones difficult to be tamed in [3+2]-cycloadditions [25], Diels-Alder- [26], and hetero-Diels-Alder-reactions [27]. Correctly, the fraction containing only the monomeric form is often described as ‘thiochalcone fraction’ [25,27]. As mentioned in the ‘Introduction’, the single experiment reporting sulfurization of a rather complex, in situ-generated thiochalcone relates to harsh reaction conditions with heating of the reaction mixture in boiling xylene [17].

The goal of the present study was to verify whether sulfurization of typical thiochalcones **3** can be performed under mild conditions using elemental sulfur activated by TBAF in THF at room temperature. The test experiment performed with **3a** and elemental sulfur (molar ratio of **3a** to [S] was 1:2) according to the protocol described above for thioketones **2a**,**b** (Entry C), was unsuccessful, however. In that case, no conversion of **3a** occurred even after three days of stirring at rt. Instead, slow decomposition of the starting **3a** was observed by TLC.

**Scheme 10:**
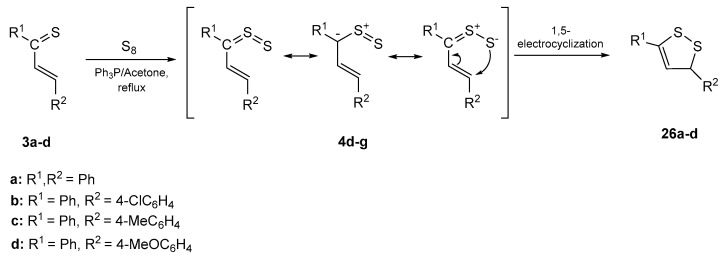
Synthesis of 3*H*-1,2-dithioles **26a**–**26d** via PPh_3_-catalyzed sulfurization of thiochalcones **3a**–**3d**.

Prompted by Okuma’s report [5], we decided to use PPh_3_ in boiling acetone solution as a nucleophilic activator of sulfur (Procedure I, Table 3). In the cited study, comparable results were obtained using either PPh_3_ or S=PPh_3_. In our hands, under these conditions a part of **3a**, consisting of monomeric thiochalcone and its dimer (dithiine derivative, underwent dissociation) reacted with sulfur yielding an orange-red colored, crystalline product. In the ^1^H-NMR spectrum (see Supplementary Materials part), along with absorptions of aromatic H atoms, a characteristic set of two doublets with ^3^*J*_H,H_ = 3.0 Hz was found at 5.87 and 6.19 ppm, respectively. In addition, ^13^C-NMR revealed a signal of a *C*_q_ atom at 63.7 ppm and two other signals located at 120.7 and 132.9 ppm, respectively, with clearly different intensities, which could be attributed to a CH=C unit. The elemental analysis confirmed the molecular formula C_15_H_12_S_2_ expected for a product of monosulfurization of **3a**. Based on the collected spectroscopic data, the structure of the obtained product was established as 3,5-diphenyl-3*H*-1,2-dithiole (**26a**, Scheme 10).

It has to be underlined that the ^1^H-NMR analysis of the crude mixture revealed the presence of the non-reacted thiopyran as one of the dimers of the starting thiochalcone [25,26,27]. Apparently, this dimer is stable under the reaction conditions and does not undergo dissociation leading to new portions of the monomeric form. This fact must be taken into account while calculating the yield of the obtained sulfurization product.

An analogous procedure has been applied to perform sulfurization of three other thiochalcones **3b**–**d**, which have also been converted into the desired 3*H*-1,2-dithioles **26b**–**d**, albeit in all cases the yields of the isolated products were rather low. The results of all experiments aimed at the synthesis of heterocycles **26** are summarized in Table 3.

For comparison reasons, two modifications of the applied procedure were tested in reactions with **3a**. In the first case, potassium thiophenolate was used as a catalyst, but after 4 h reaction time the product **26a** was isolated in lower yield (15%) (Procedure II). The second modification comprised replacement of acetone by higher boiling butanone, and in this case, the reaction was finished after only 30 min, yielding **26a** in the comparable yield of 41% (Procedure III, Table 3).

The alternative procedure with pre-dried CsF in DMF solution (Procedure IV) was also tested in reactions with **3a** and **3c**. After 3d at rt the reactions were finished and the ^1^H-NMR analysis of the crude mixtures indicated the presence of the expected 3*H*-1,2-dithioles **26a** and **26c**, which were isolated by column chromatography in 15% and 17% yields, respectively.

Notably, no formation of a corresponding 1,2,4-trithiolane was observed in any experiment performed with thiochalcones **3**.

A feasible reaction mechanism is presented in Scheme 10. The PPh_3_ catalyzed sulfurization of thiochalcones involves α,β-unsaturated thiocarbonyl *S*-sulfides **4** as plausible intermediates. They are formed via sulfur transfer from in situ-generated, neutral (and therefore less nucleophilic) triphenylphosphine polysulfides to the C=S group. In reactions catalyzed with CsF (Procedure IV), participation of fluoropolysulfide anion **8B** in the transfer of the sulfur atom is likely. In that case, however, the intermediate dithiirane of type **21**, derived from the respective thiochalcone **3**, is belived to undergo ring opening along the C–S bond yielding the respective 1,3-dipole [22,23,28]. Thiocarbonyl *S*-sulfides derived from chalcones belong to the group of α,β-unsaturated 1,3-dipoles and, therefore, they can undergo 1,5-electrocyclization [29] leading to the formation of 3*H*-1,2-dithioles, which apparently is a faster intramolecular process than the competitive [3+2]-cycloaddition with a ‘non-sulfurized’ molecule of the starting thiochalcone. Therefore, in none of the experiments formation of a respective 1,2,4-trithiolane was observed. Participation of thiocarbonyl *S*-sulfides in the [3+2]-cycloaddition with thiocarbonyl dipolarophiles is an important method for the synthesis of sulfur-rich heterocycles and diverse methods for the generation of this type of ‘sulfur-centered 1,3-dipoles’ are known [28]. Most importantly, the described 1,5-dipolar electrocyclization found for the first time in sulfurization reactions of thiochalcones offers a convenient method for the preparation of useful, five-membered 3*H*-1,2-dithioles **26**.

## 3. Materials and Methods

### 3.1. Materials

Elemental sulfur (S_8_) in the form of a powder (99.98% purity), molecular sieves 4Å (mesh 8–12), tetrabutylammonium fluoride (TBAF, 1.0 M solution in THF) and benzophenone (99%) were purchased from Merck (Sigma-Aldrich, Darmstadt, Germany). Triphenylphosphine (Ph_3_P) (>95%), cesium fluoride (CsF) and 1,1,2,2-tetrachloroethane, used as internal standard in NMR analysis, were received from Tokyo Chemical Industry (TCI, Tokyo, Japan). Organic solvents were purchased from the following companies: tetrahydrofuran (99.9%) from Chemsolve (Witko, Lodz, Poland), dichloromethane (99.9%) from Honeywell (Charlotte, NC, USA), anhydrous *N,N*-dimethylformamide (99.8%) from Merck (Sigma-Aldrich), methanol (99.8%), acetone (99.5%) and petroleum ether 40/60 from Chempur (Piekary Śląskie, Poland). THF was dried over sodium (Na) with an addition of benzophenone, and acetone was distilled before its usage. Starting thioketones were obtained following reported procedures: thiobenzophenone (**1b**) from benzophenone by treatment with Lawesson’s reagent in toluene solution [18]; 2,2,4,4-tetramethylcyclobutane-1,3-dithione (‘dithione’) (**2a**), 2,2,4,4-tetramethyl-3-thioxocyclo-butanone(‘monothione’) (**2b**), and admantanthione (**2c**) from 2,2,4,4-tetramethylcyclobutane-1,3-dione (**13**) and adamantane-2-one, respectively, by treatment with P_4_S_10_ in pyridine solution [18]. Thiochalcones **3** were prepared from the corresponding chalcones by thionation with Lawesson’s reagent and subsequently purified by column chromatography on silica gel [25,26].

### 3.2. Analytical Methods and Equipment

The nuclear magnetic resonance (NMR) spectra were measured using a Bruker Avance III instrument (Bruker, Billerica, MA, USA) at 600 MHz (^1^H-NMR) and at 151 MHz (^13^C-NMR), respectively. Unless otherwise indicated the measurements were carried out in deuterochloroform (CDCl_3_), using solvent signals as a reference (^1^H-NMR, δ = 7.26 ppm (CDCl_3_); ^13^C-NMR, δ = 77.0 ppm (CDCl_3_)). Chemical shifts (δ) were presented in ppm and coupling constants (*J*) were calculated in Hz. Integrals are given in accordance with assignments. Melting points were determined in capillaries with a Melt-Temp II apparatus (Laboratory Devices, Holliston, MA, USA). Elemental analyses were obtained with a Vario EL III instrument (Elementar Analysensysteme, Langenselbold, Germany).

### 3.3. Conversions of Thioketones **2a**,**2b** in the Presence of Fluoride Anion and Absence of S_8_

#### 3.3.1. Procedure I: TBAF/THF

To a stirred solution of pre-dried TBAF (0.1 mL of 1.0 M solution in THF) in 1.5 mL of dried THF, 0.5 mmol of the corresponding thioketone **2a** or **2b** was added at rt under Ar atmosphere. The solution was stirred and after ca. 5 min the color changed from rubin-red (**2a**) or orange-red (**2b**) to yellow-orange. Next, the solvent was evaporated in vacuo and the residue was pre-purified by filtration via a short chromatographic column filled with SiO_2_ (*ca*. 2 cm layer). Crude mixtures were analyzed in the ^1^H-NMR spectra with weighted standard (Cl_2_CH–CHCl_2_). The products formed were identified by comparison of their spectroscopic data with reported ones. Yields reported below refer to the analysis of crude mixtures performed with weighted standard (method A) or to amounts of isolated products (method B).

#### 3.3.2. Procedure II: CsF/DMF

In an alternative procedure, conversion of thioketone **2b** was carried out in dry DMF (1.5 mL) using cat. amounts of freshly calcined cesium fluoride at rt under Ar atmosphere. After 2 h of magnetic stirring, the color still remained orange-red and no formation of other products was observed by TLC control. After that time, a drop of water was added to the stirred solution and the mixture rapidly changed the color to orange indicating thereby conversion of **2b**. The reaction solution was diluted with a portion of CH_2_Cl_2_ (5 mL) and extracted four times with water (4 mL each). Collected organic fractions were dried over MgSO_4_, filtered, and the solvent was evaporated. The mixture of crude products was analyzed in the ^1^H-NMR spectrum with weighted standard (Cl_2_CH–CHCl_2_); the yields of identified products **13**, **15**, and **16b** were determined by method A.

#### 3.3.3. Product Characterization

*3,3-Dimethyl-4-(propan-2-ylidene)thietane-2-thione* (**14**). Yield: Procedure I—92% (method A); 69 mg (80%) (method B). Yellow oil (ref. [18]). ^1^H-NMR: δ 1.47 (s, 2CH_3_), 1.73 (s, CH_3_), 1.86 (s, CH_3_) ppm. ^13^C-NMR: δ 20.1 (CH_3_), 21.9 (CH_3_), 25.2 (2CH_3_), 74.2 (C-3), 122.6 (=C), 131.0 (=C), 243.5 (C=S) ppm.

*2′,2′,4′,4′-Tetramethyl-3′-oxocyclobutyl 2,2,4-trimethyl-3-oxopentanedithioate (***15***)*. Yield: Procedure I—59% (method A), 38 mg (48%) (method B); Procedure II—44% (method A). Yellow solid, m.p. 57–58 °C (ref. [18], m.p. 62–63 °C). ^1^H-NMR: δ 1.13 (d, *J* = 6.6 Hz, 2CH_3_(CH)), 1.21 (s, 2CH_3_), 1.47 (s, 2CH_3_), 1.68 (s, 2CH_3_), 2.91 (sept., *J* = 6.6 Hz, 2CH_3_(CH)), 4.25 (s, HC-1′) ppm. ^13^C-NMR: δ 20.4 (2CH_3_), 21.8 (2CH_3_), 25.0 (2CH_3_), 26.8 (2CH_3_), 37.0 (C-4), 57.7 (C-2′, C-4′), 61.1, 69.4 (C-1′, C-2), 212.0 (C=O, C-3), 218.9 (C=O, C-3′, cyclobutanone), 241.2 (C=S) ppm. C_16_H_26_O_2_S_2_ (314.51): calculated, C 61.10, H 8.33, S 20.39; found, C 61.12, H 8.44, S 20.50.

*3,3,5,5-Tetramethyl-2-thioxothiolan-4-one (***16b***)*. Yield: Procedure I—13% (method A); 9 mg (10%) (method B); Procedure II: 11% (method A). Yellow thick oil (ref. [4], mp 39–41 °C). ^1^H-NMR: δ 1.44 (s, 2CH_3_), 1.70 (s, 2CH_3_) ppm. ^13^C-NMR: δ 27.8, 28.1 (2CH_3_ each), 61.3 (C-3), 63.4 (C-5), 216.7 (C=O), 234.1 (C=S) ppm.

*2,2,4,4-Tetramethylcyclobutane-1,3-dione* (**13**). Yield: Procedure I—12% (method A); Procedure II—8% (method A). ^1^H-NMR: δ 1.34 ppm. (ref. [30]).

### 3.4. General Procedures of the Fluoride Anion Catalyzed Sulfurization of Thioketones **1** and **2** with Elemental Sulfur (S_8_)

#### 3.4.1. Procedure I: TBAF/THF

To the suspension of elemental sulfur (calculated on atomic [S]) (0.25 mmol, Entry A; 0.50 mmol, Entry B; 1.00 mmol, Entry C, 2.00 mmol, Entry D, or 4.00 mmol, Entry E) in 1.5 mL of dried THF, under argon atmosphere, 0.1 mL (0.1 mmol, 20% mol. calculated on the amounts of thioketone used) of the tetrabutylammonium fluoride solution (TBAF, 1.0 M in THF), pre-dried over molecular sieves, was added dropwise. The mixture became red (Entries B–D)) or olive green in color (Entry A) and the sulfur has dissolved forming a homogenous solution. After ca. 10–15 min of magnetic stirring at rt, 0.5 mmol of the corresponding thioketone **1a** or **2a**,**2c** was added in small portions to the solution and stirring was continued for an appropriate time at rt (see Table 1 and Table 2). Next, the solvent was evaporated in vacuum and the residue was pre-purified by filtration via a short chromatographic column filled with SiO_2_ (*ca.* 2 cm layer). The crude mixtures were analyzed in the ^1^H-NMR spectra with weighted standard (Cl_2_CH–CHCl_2_) and subsequently separated on preparative chromatographic plates coated with silica gel. A mixture of petroleum ether and dichloromethane (in most cases the ratio of both solvents was 9:1 or 8:2) was used as an eluent. Yields refer either to analysis with weighted standard (method A) or to isolated amounts (method B).

#### 3.4.2. Procedure II: CsF/DMF

In an alternative procedure reactions of thioketones **1a** and **2a**–**c** with 2.0 mmol of sulfur (Entry C′) were carried out in dry DMF (2–3 mL) using catalytic amounts of freshly calcined cesium fluoride (CsF). After completion of the reactions, the mixture was diluted with dichloromethane and extracted several times with small portions of water to remove DMF. Following an alternative method for the removal of DMF, toluene (2–3 mL) was added in small portions and the solutions obtained thereby were warmed in a water bath (55 °C) to evaporate the solvents. Crude products were purified by column chromatography on silica gel using petroleum ether/dichloromethane mixtures as eluent.

#### 3.4.3. Product Characterization

*Dispiro[adamantane-2,3′-(1,2,4)-trithiolane-5′,2″-adamantane] (***5c***)*. Yields: Procedure I—(a) Entry A: 27 mg (59% based on S/30% based on **1c**); (b) Procedure II—Entry C′: 54 mg (59%) (method B). Colorless solid, m.p. 200–201 °C (methanol/CH_2_Cl_2_) (ref. [31], m.p. 191–192 °C). ^1^H-NMR: δ 1.75–1.95 (m, 16H), 2.15–2.40 (m, 12H) ppm. ^13^C-NMR: δ 26.6, 26.9 (4CH), 36.7, 37.2, 37.9 (10CH_2_, ratio ca. 2:2:1), 39.4 (4CH), 90.3 (C-3′, C-5′) ppm.

*3,3,6,6-Tetraphenyl-1,2,4,5-tetrathiane (***6a***)*. Yields: Procedure I—(a) Entry B: 32 mg (28%) (method B); (b) Entry C: 60 mg (52%) (method B); (c) Procedure II—Entry C′: 65 mg (56%) (method B). Colorless crystals, m.p. 210–212 °C (decomposition, blue) (methanol/CH_2_Cl_2_) (ref. [3], mp. 209–209.5 °C). ^1^H-NMR: δ 7.30–7.36 (m, 12CH_arom_.), 7.55–7.59 (m, 8CH_arom_.) ppm. ^13^C-NMR: δ 71.4 (C-3, C-6), 127.9, 128.5, 128.6 (20CH_arom_.), 141.7 (4C_arom_.) ppm.

*1,1,3,3,8,8,10,10-Octamethyl-5,6,11,12-tetrathiadispiro-[3.2.3.3]dodecane-2,9-dione (***6b***)*. Yields: Procedure I—(a) Entry A: 12% (method A); (b) Entry B: 40% (method A), 30 mg (32%) (method B). Colorless solid, m.p. 170–172 °C (methanol/CH_2_Cl_2_) (ref. [4], m.p. 169–170 °C). ^1^H-NMR: δ 1.39 (s, br, 4CH_3_), 1.54 (s, br, 4CH_3_) ppm. ^13^C-NMR: δ 20.9, 24.9 (4CH_3_ each), 67.1 (C-1, C-3, C-8, C-10), 71.5 (C-4, C-7), 217.9 (2C=O) ppm.

*1,1,3,3,8,8,10,10-Octamethyl-5,6,11,12-tetrathiadispiro-[3.2.3.3]-9-oxododecane-2-thione* (**6e**). Yield: Procedure I—(a) Entry B: 15 mg (15%) (method B). Orange oil. ^1^H-NMR: δ 1.42 (s, br, 4CH_3_), 1.69 (s, br, 4CH_3_) ppm. ^13^C-NMR: δ 27.8, 28.1 (4CH_3_ each), 61.3 (C-1, C-3), 63.4 (C-8, C-10), 77.86, 77.91 (C-4, C-7), 216.6 (C=O), 243.0 (C=S) ppm.C_16_H_24_OS_5_ (392.69): calculated, C 48.94, H 6.16, S 40.83; found, C 48.96, H 6.33, S 40.58.

*1,1,3,3,8,8,10,10-Octamethyl-5,6,11,12,13-pentathiadispiro-[3.2.3.3]tridecane-2,9-dione (***7a***)*. Yields: Procedure I—(a) Entry C: 35 mg (34%) (method B); (c) Entry D: 53 mg (52%) (method B); (d) Entry E: 61 mg (60%) (method B); Procedure II—(e) Entry C′: 44 mg (43%) (method B). Colorless crystals, m.p. 146–148 °C (methanol/CH_2_Cl_2_) (ref. [4,22], m.p. 147–148 °C). ^1^H-NMR: δ 1.20–1.90 (m, 8CH_3_) ppm. ^13^C-NMR: δ 21.0–26.0 (m, 8CH_3_), 67.3 (C-1, C-3, C-8, C-10), 71.5 (C-4, C-7), 216.2 (2C=O) ppm. C_16_H_24_O_2_S_5_ (408.69): calculated, C 47.02, H 5.92, S 39.23; found, C 47.13, H 6.07, S 39.34.

*Dispiro[adamantane-2,4′-(1,2,3,5,6)-pentathiepane-7′,2”-adamantane] (***7b***)*. Yields: Procedure I—(a) Entry A: 20 mg (37% based on S/19% based on **2c**) (method B); (b) Entry B: 50 mg (47%) (method B); (c) Entry C: 55 mg (51%) (method B). Colorless solid, m.p. > 236 °C (decomposition) (methanol/CH_2_Cl_2_) (ref. [3]; m.p. > 236 °C, decomp.). ^1^H-NMR: δ 1.55–3.0 (m, 28H) ppm. ^13^C-NMR: δ 27.1, 33.7, 34.1, 35.4, 37.6, 38.6 (18C), referred weak signal at 84 ppm (C-4′, C-7′) is missing (see: ref. [3]).

*1,1,3,3,8,8,10,10-Octamethyl-5,6,11,12,13-pentathiadispiro-[3.2.3.3]tridecane-2,9-dithione (***7c***)*. Yields: Procedure I—(a) Entry C: 25 mg (23%) (method B); (b) Entry D: 51 mg (46%) (method B); Procedure II—(c) Entry C′: 59 mg (54%) (method B); red-orange viscous oil. ^1^H-NMR: δ 1.30–2.00 (m, 8CH_3_) ppm; ^1^H-NMR (CCl_2_=CCl_2_, D_2_O, 90 °C): δ 1.92, 1.98, 2.08, 2.20 (4s, br, 2CH_3_ each) ppm. ^13^C-NMR: δ 24.0–29.0 (m, 8CH_3_), 50.9 (C-1, C-3, C-8, C-10) 53.5 (C-4, C-7), 276.0 (2C=S) ppm. C_16_H_24_S_7_ (440.82): calculated, C 43.59, H 5.49, S 50.92; found, C 43.37, H 5.38, S 50.97.

*3,3-Dimethyl-4-(propan-2-ylidene)thietane-2-thione (***14***)*. Yields: Procedure I—(a) Entry A: 63% (method A); (b) Entry B: 9% (method A); (c) Entry C: 3% (method A).

*2′,2′,4′,4′-Tetramethyl-3′-oxocyclobutyl 2,2,4-trimethyl-3-oxopentanedithioate (***15***)*. Yields: Procedure I—(a) Entry A: yield: 30% (method A); (b) Entry B: 20% (method A).

*3,3,5,5-Tetramethylthiolane-2,4-dithione (***16a***)*. Yields: Procedure I—(a) Entry A: 11% (method A); (b) Entry B: 8 mg (8%) (method B); (c) Entry C: 8% (method A), 8 mg (8%) (method B); (d) Entry D: 5% (method A); 3 mg (3%) (method B). Red-orange oil (ref. [4]: b.p. 122 °C). ^1^H-NMR: δ 1.61 (s, 2CH_3_), 1.83 (s, 2CH_3_) ppm. ^13^C-NMR: δ 33.5 (2CH_3_), 33.7 (2CH_3_), 71.0 (C-3), 77.8 (C-5), 245.3 (S=C-2), 267.4 (S=C-4) ppm.

*3,3,5,5-Tetramethyl-2-thioxothiolane-4-one (***16b***)*. Yields: (a) Entry A: 10% (method A); (b) Entry B: 23% (method A), 16 mg (17%) (method B).

1,1,4,4,8,8,11,11-Octamethyl-2,6,9,12-tetrathiadispiro[4.1.4.1]dodecane-3,10-dithione (meso-**17**), and 1,1,4,4,8,8,11,11-Octamethyl-2,6,10,12-tetrathiadispiro[4.1.4.1]dodecane-3,9-di-thione (d,l-**17**). Yield: Procedure I—(a) Entry B: 44 mg (43%) (method B). Yellow crystals, m.p. 130–138 °C (methanol/CH_2_Cl_2_). ^1^H-NMR: δ 1.32 (s, 2CH_3_), 1.35 (s, 2CH_3_), 1.51 (s, 2CH_3_), 1.51 (s, 2CH_3_), 1.69 (s, 2CH_3_), 1.71 (s, 2CH_3_), 1.74 (s, 2CH_3_), 1.75 (s, 2CH_3_) ppm. ^13^C-NMR: δ 26.32 (2CH_3_), 26.36 (2CH_3_), 26.57 (2CH_3_), 26.61 (2CH_3_), 29.72 (2CH_3_), 29.80 (2CH_3_), 30.66 (4CH_3_), 58.20 (2C), 58.57 (2C), 64.22 (2C), 64.51 (2C), 78.54 (2C-5), 78.71 (2C-7), 248.18 (2C=S), 248.39 (2C=S) ppm. C_16_H_24_S_6_ (408.75): calculated, C 47.01, H 5.92, S 47.07; found, C 46.91, H 6.15, S 46.81.

*4,4-Dimethyl-5-(propan-2-ylidene)-1,2-dithiolane-3-thione (***18**). Yields: Procedure I—(a) Entry C: yield: 5% (method A); (b) Entry D: yield: 2% (method A). Yellow-orange oil (ref. [21], yellow oil). ^1^H-NMR: δ 1.66 (s, 2CH_3_), 1.99 (s, CH_3_), 2.02 (s, CH_3_) ppm. ^13^C-NMR: δ 21.4 (CH_3_), 26.4 (CH_3_), 29.6 (2CH_3_), 64.2 (C-4), 128.2 (=C), 137.10 (=C), 247.9 (C=S) ppm.

*1,1,3,3-Tetramethyl-5,6,7,8,9,10-hexathiaspiro[3.6]decane-2-thione (***19***)*. Yield: Procedure I—(a) Entry D: 7 mg (4%) (method B). Pink solid, m.p. 111‒114 °C (MeOH/CH_2_Cl_2_). ^1^H-NMR: δ 1.54 (s, br, 4CH_3_) ppm. ^13^C-NMR: δ 22.5, 28.6 (2CH_3_ each), 70.6 (C-1, C-3), 95.6 (C-4), 274.2 (C=S) ppm. C_8_H_12_S_7_ (332.64): calculated, C 28.89, H 3.64, S 67.48; found, C 29.11, H 3.70, S 67.18.

### 3.5. General Procedures for Sulfurization of Thiochalcones **3** with Elemental Sulfur (S_8_)

#### 3.5.1. Procedure I: Ph_3_P/Acetone

In a two-necked round-bottomed flask (10 mL) equipped with a magnetic stirrer 0.5 mmol of the corresponding thiochalcone **3a**–**d**, 0.125 mmol of elemental sulfur (S_8_) (1 mmol calculating on atomic [S]) and 0.125 mmol of triphenylphosphine (Ph_3_P) were placed and 2 mL of dry acetone were added. The resulting mixture was heated to the reflux under condenser and under argon and stirred for the appropriate time (discoloring). Then, the solvent was evaporated in vacuo and the residue was purified, firstly by short column chromatography and afterwards by thin-layer chromatography, by using SiO_2_ as the absorbent and a mixture of petroleum ether and dichloromethane (9:1) as the eluent, yielding the corresponding 3*H*-1,2-dithiole derivative **26a**–**d**. Reported yields refer either to isolated amounts of products **26** (Method A) or to amounts calculated on the basis of standard analysis performed with weighted amounts of Cl_2_CH–CHCl_2_ added to the crude mixture (method B).

#### 3.5.2. Procedure II: PhSK/Acetone

In analogy to Procedure I, thiochalcone **3a** was reacted with elemental sulfur in the presence of cat. amounts (0.06 mol-equiv.) of potassium thiophenolate (PhSK) in boiling acetone (see ref. [3,4]).

#### 3.5.3. Procedure III: Ph_3_P/Butanone

In analogy to Procedure I, thiochalcone **3a** was reacted with elemental sulfur in the presence of cat. amounts of PPh_3_ using butanone as a solvent.

#### 3.5.4. Procedure IV: CsF/DMF

Thiochalcones **3a** or **3c**, (1 mmol) and 64 mg (2.0 mmol) of sulfur were stirred magnetically in dry DMF (2.5 mL) with cat. amounts of freshly calcined cesium fluoride (CsF). After 72h the reaction solution was diluted with dichloromethane (25 mL) and extracted several times with small portions of water for removal of DMF. Combined organic layers were dried over MgSO_4_, filtered and purified by column chromatography on silica gel using a 95:5 mixture of petroleum ether and dichloromethane as eluent.

#### 3.5.5. Product Characterization

*3,5-Diphenyl-3H-1,2-dithiole* (**26a**). Yields: (a) Procedure I—51 mg (40%) (method A), 63% (method B); (b) Procedure II—53 mg (41%) (method A); (c) Procedure III—19 mg (15%) (method A); Procedure IV: 38 mg (15%) (method A). Orange-red solid, m.p. 78–81 °C. ^1^H-NMR: δ 5.87 (d, *J* = 3.0 Hz, 1H, CH); 6.19 (d, *J* = 3.0 Hz, 1H, CH); 7.34–7.38 (m, 1H, CH_arom_.); 7.39–7.44 (m, 5H, CH_arom_.); 7.49–7.53 (m, 2H, CH_arom_.); 7.57–7.61 (m, 2H, CH_arom_.) ppm. ^13^C-NMR: δ 63.7 (C-3); 120.7 (C-4); 127.4, 127.5, 128.4, 128.8 129.0, 129.3 (10C_arom_.); 132.9 (C-5); 140.8, 145.2 (2C_arom_.) ppm. C_15_H_12_S_2_ (256.39): calculated, C 70.27, H 4.72, S 25.01; found: C 70.20, H 4.68, S 25.01.

*3-(4-Chlorophenyl)-5-phenyl-3H-1,2-dithiole* (**26b**). Yield: (a) Procedure I—22 mg (15%) (method A). Red oil. ^1^H-NMR: δ 5.80 (d, *J* = 3.6 Hz, 1H, CH); 6.15 (d, *J* = 3.6 Hz, 1H, CH); 7.36 (d, *J* = 9.0 Hz, 2H, CH_arom_.), 7.39–7.45 (m, 5H, CH_arom_.), 7.56–7.59 (m, 2H, CH_arom_.) ppm. ^13^C-NMR: δ 62.7 (C-3); 120.1 (C-4); 127.4, 128.8, 129.1, 129.4 (9C_arom_.); 132.7 (C-5); 134.2, 139.4, 145.7 (3C_arom_.) ppm. C_15_H_11_S_2_Cl (290.83): calculated, C 61.95, H 3.81, S 22.05; found: C 61.68, H 3.85, S 22.01.

*3-(4-Methylphenyl)-5-phenyl-3H-1,2-dithiole* (**26c**). Yield: (a) Procedure I—27 mg (20%) (method A); Procedure IV—46 mg (17%) (method A). Red solid, m.p. 79–82 °C. ^1^H-NMR: δ 2.39 (s, 3H, CH_3_); 5.86 (d, *J* = 3.3 Hz, 1H, CH); 6.18 (d, *J* = 3.3 Hz, 1H, CH); 7.21, 7.59 (2d, *J* = 8.4 Hz, 4H, AB system), 7.38–7.42 (m, 5H, CH_arom_.) ppm. ^13^C-NMR: δ 21.2 (CH_3_), 63.5 (C-3), 120.9 (C-4), 127.4, 128.7, 129.2, 129.6, (9C_arom_.); 133.0 (C-5), 137.8 138.3, 145.0 (3C_arom_.) ppm. C_16_H_14_S_2_ (270.41): calculated, C 71.07, H 5.22, S 23.72; found: C 70.92, H 5.43, S 23.61.

*3-(4-Methoxyphenyl)-5-phenyl-3H-1,2-dithiole* (**26d**). Yield: (a) Procedure I—17 mg (12%) (method A). Red oil. ^1^H-NMR: δ 3.84 (s, 3H, OCH_3_); 5.87 (d, *J* = 3.6 Hz, 1H, CH); 6.17 (d, *J* = 3.6 Hz, 1H, CH); 6.93 (d, *J* = 9.0 Hz, 2H, CH_arom_.), 7.38–7.44 (m, 5H, CH_arom_.), 7.57–7.60 (m, 2H, CH_arom_.) ppm. ^13^C-NMR: δ 55.4 (OCH_3_), 63.3 (C-3), 114.3 (C-4), 121.0, 127.4, 127.7, 127.8, 129.21 (9C_arom_.); 132.9 (C-5), 133.0, 144.8, 159.7 (3C_arom_.) ppm.

## 4. Conclusions

The presented study showed that fluoride anion can be applied as an excellent activator of elemental sulfur in sulfurization reactions of easily available cycloaliphatic thioketones derived from 2,2,4,4-tetramethylcyclobutane-1,3-dione. Depending on the molar ratio of the starting materials, sulfur-rich heterocycles with variable ring size can be obtained in satisfactory to good yields. In situ-generated fluoropolysulfide anions act as powerful sulfurizing reagents. As a key intermediate in the studied reactions, three-membered, reactive dithiiranes, formed via the addition of the latter to the C=S group, are postulated for both cycloaliphatic and aromatic thioketones.

In contrast to thioketones, their α,β-unsaturated analogues, i.e., thiochalcones, do not undergo the sulfurization reaction with sulfur in the presence of fluoride anion. On the other hand, their sulfurization with elemental sulfur, leading to 3*H*-1,2-dithiole derivatives, can be performed using triphenylphosphine (PPh_3_) as a catalyst. The latter heterocycles are of interest as unique starting materials for the preparation of sulfur-heterocycles based persistent radicals [32,33]. However, up to now, methods for the synthesis of 3*H*-1,2-dithioles and their conversion into the stable organic radicals are rarely reported and they are described only in rather remote literature [34].

The developed protocols of sulfurization in the presence of fluoride anion can be of interest not only for the synthesis of sulfur-rich heterocycles, such as 1,2,4-trithiolanes [28], 1,2,4,5-tetrathianes [35,36] or 1,2,3,5,6-pentathiepanes, but also for the preparation of sulfur-rich polymers starting with alkyl- [37,38] or aryl [39] substituted thiiranes. In the present work, fluoride anion was shown to act as an alternative, nucleophilic catalyst and can replace smelly and unstable sodium (or potassium) thiophenolate in the activation of elemental sulfur in reactions with thiiranes. The presented research should be considered as a new contribution to our continuous studies on exploration of elemental sulfur and small-ring, congested *S*-heterocycles (thiaziridiens, thiiranes) in sulfurization reactions of diverse organic compounds, including stable thioketones [10,22,24,30] but also transient nucleophilic heterocyclic carbenes (NHC) [40,41].

## Figures and Tables

**Table 1:** Sulfurization conditions of thioketones **2a** and **2b** and yields of the products.

Thioketone	Entry	RT [h], rt	Product	Yield (%)
**2a**	A	1	**14**	63 ^a^
			**16a**	11 ^a^
	B	1.5	**16a**	8 ^b^
			**6e**	15 ^b^
			***meso*** **-17,** **D,L** **-17**	43 ^b^
	C	2	**14**	9 ^a^
			**16a**	8 ^b^
			**18**	5 ^a^
			**7c**	23 ^b^
	C′	24	**7c**	54 ^b^
	D	2	**14**	3 ^a^
			**16a**	5 ^a^
			**18**	2 ^a^
			**7c**	46 ^b^
			**19**	4 ^b^
**2b**	A	1.5	**15**	30 ^a^
			**16b**	10 ^a^
			**6b**	12 ^a^
	B	3	**15**	20 ^a^
			**16b**	23 ^a^
			**6b**	40 ^a^
	C	48	**7a**	34 ^b^
	C′	24	**7a**	43 ^b^
	D	22	**7a**	52 ^b^
	E	6	**7a**	60 ^b^

^a^ Method A (^1^H-NMR); ^b^ Method B (isolated products).

**Table 2:** Sulfurization conditions of thioketones **2c** and **1b** and yields of the products (isolated amounts).

Thioketone	Entry	RT (h)		Product	Yield (%)		Product	Yield (%)
**2c**	A	72	**5c**		59	**7b**		37
	B	46	-		-	**7b**		47
	C	24	-		-	**7b**		51
	C′	24	**5c**		59	-		-
**1a**	B	72	**6a**		28	-		-
	C	72	**6a**		52	-		-
	C′	48	**6a**		56	-		-

**Table 3:** Sulfurization conditions of thiochalcones **3a**–**3d** and yields of products.

Thiochalcone	Procedure	RT [h]	Product	Yield (%)
**3a**	I	3.5	**26a**	40 ^a^ (63 ^b^)
**3a**	II	4.0	**26a**	15 ^a^
**3a**	III	0.5	**26a**	41 ^a^
**3a**	IV	72	**26a**	15 ^a^
**3b**	I	4.0	**26b**	15 ^a^
**3c**	I	3.5	**26c**	20 ^a^
**3c**	IV	72	**26c**	17 ^a^
**3d**	I	3.0	**26d**	12 ^a^

^a^ Method A (isolated product); ^b^ Method B (^1^H-NMR).

## Data Availability

All data supporting the presented study are included into the Supplementary Material part.

## Supplementary Materials

The Supplementary Materials are available online and they contain the scanned ^1^H- and ^13^C-NMR spectra for the described and isolated compounds. The re-determined X-ray crystallography data for compound **7a** are deposited as CSD Communication under deposition number 2056867 (doi:10.5517/ccdc.csd.cc271bk6).

## Sample Availability

Samples of the compounds **5a**, **6a**,**b**,**e**, **7a**‒**7c**, **13**‒**15**, **16a**‒**16b**, *meso*-**17**, *d,l*-**17**, **19**, and **26a**‒**26d** are available from the authors.
