# Supplementary material for: The Fluoride Anion-Catalyzed Sulfurization of Thioketones with Elemental Sulfur Leading to Sulfur-Rich Heterocycles: First Sulfurization of Thiochalcones
Source: Molecules. 2021 Feb 5;26(4):822. doi: 10.3390/molecules26040822 (PMC7914474; doi:10.3390/molecules26040822)
Supplement: Supplementary file 1 [file molecules-26-00822-s001.pdf]

molecules

Supplementary materials

The Fluoride Anion-Catalyzed Sulfurization of Thioketones with Elemental Sulfur Leading to Sulfur-Rich Heterocycles: First Sulfurization of Thiochalcones †

Grzegorz Mlostoń [1,*], Jakub Wręczycki [2,*], Katarzyna Urbaniak [1], Dariusz M. Bieliński [2] and Heinz Heimgartner [3]

[1] Department of Organic and Applied Chemistry, Faculty of Chemistry, University of Lodz, 12 Tamka Street, 91-403 Lodz, Poland; katarzyna.urbaniak@chemia.uni.lodz.pl
[2] Institute of Polymer and Dye Technology, Faculty of Chemistry, Lodz University of Technology, 12/16 Stefanowskiego Street, 90-924 Lodz, Poland; dariusz.bielinski@p.lodz.pl
[3] Department of Chemistry, University of Zurich, Winterthurerstrasse 190, CH-8057 Zurich, Switzerland; heinz.heimgartner@chem.uzh.ch
* Correspondence: grzegorz.mloston@chemia.uni.lodz.pl (G.M.); jakub.wreczycki@dokt.p.lodz.pl (J.W.); Tel.:+48-42-635-57-61 (G.M.)
† Dedicated to Professor Janusz Jurczak (Warsaw) on the occasion of his 80th birthday.

Content:

- Copies of ^{1}H-NMR and ^{13}C-NMR spectra of synthesized compounds
- Copies of selected ^{1}H-NMR spectra of crude mixtures weighted with internal standard

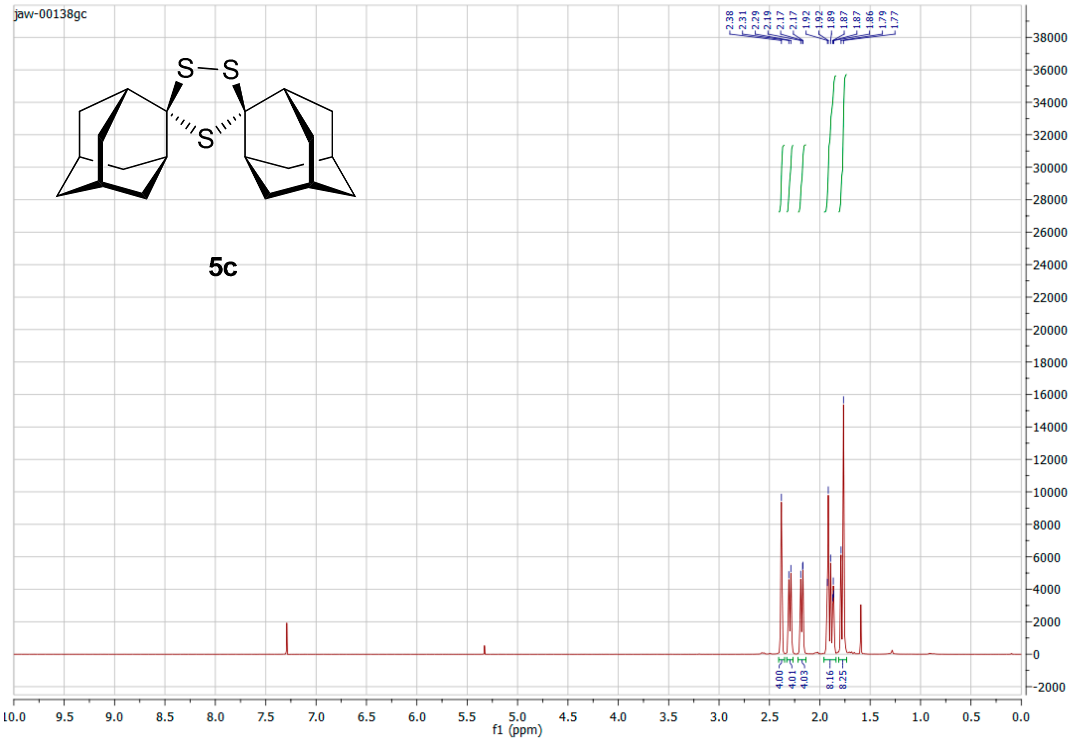

Figure S1. ^{1}H-NMR of dispiro[adamantane-2,3'-(1,2,4)-trithiolane-5',2''-adamantane] (**5c**): ($CDCl_3$, 600 MHz).

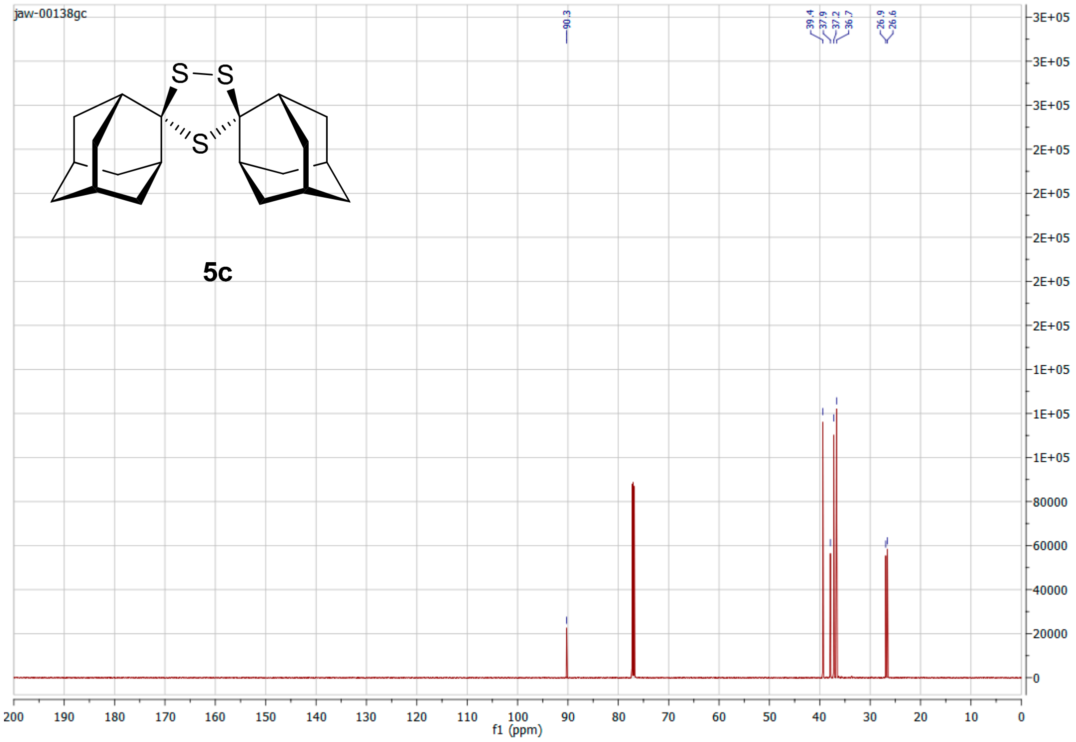

Figure S2. ^{13}C-NMR of dispiro[adamantane-2,3'-(1,2,4)-trithiolane-5',2''-adamantane] (**5c**): ($CDCl_3$, 151 MHz).

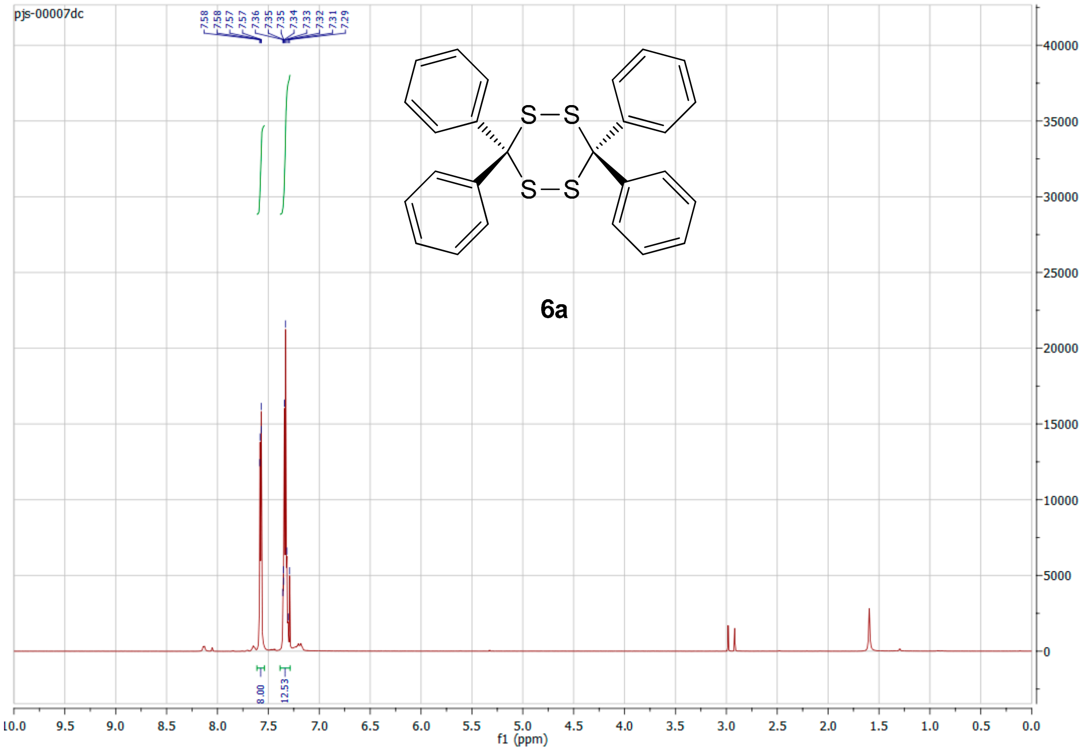

Figure S3. ^{1}H-NMR of 3,3,6,6-tetraphenyl-1,2,4,5-tetrathiane (**6a**) ($CDCl_3$, 600 MHz).

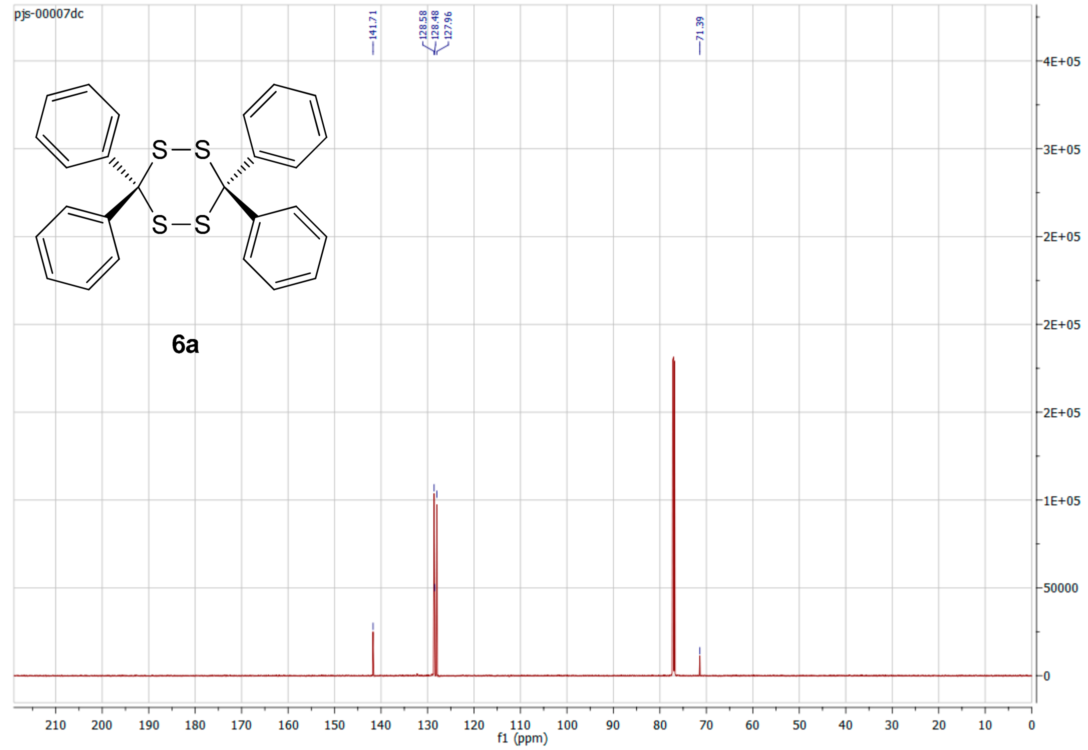

Figure S4. ^{13}C-NMR of 3,3,6,6-tetraphenyl-1,2,4,5-tetrathiane (**6a**) ($CDCl_3$, 151 MHz).

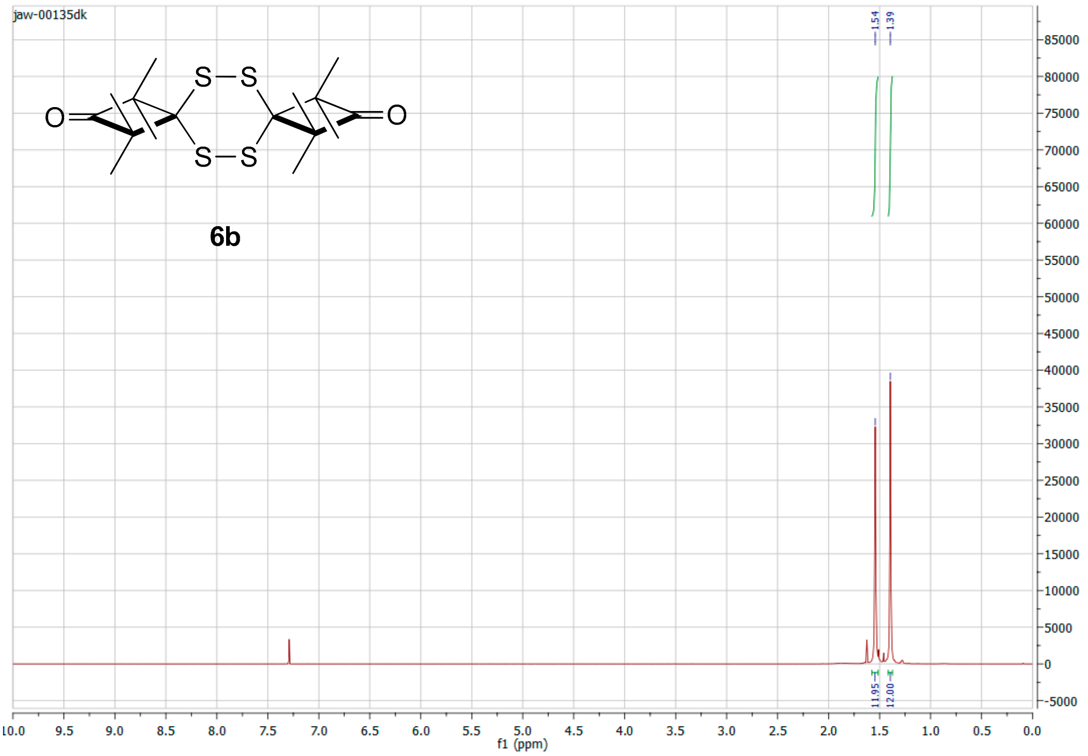

Figure S5. ^{1}H-NMR of 1,1,3,3,8,8,10,10-octamethyl-5,6,11,12-tetrathiadispiro-[3,2,3,3]dodecane-2,9-dione (**6b**) ($CDCl_3$, 600 MHz).

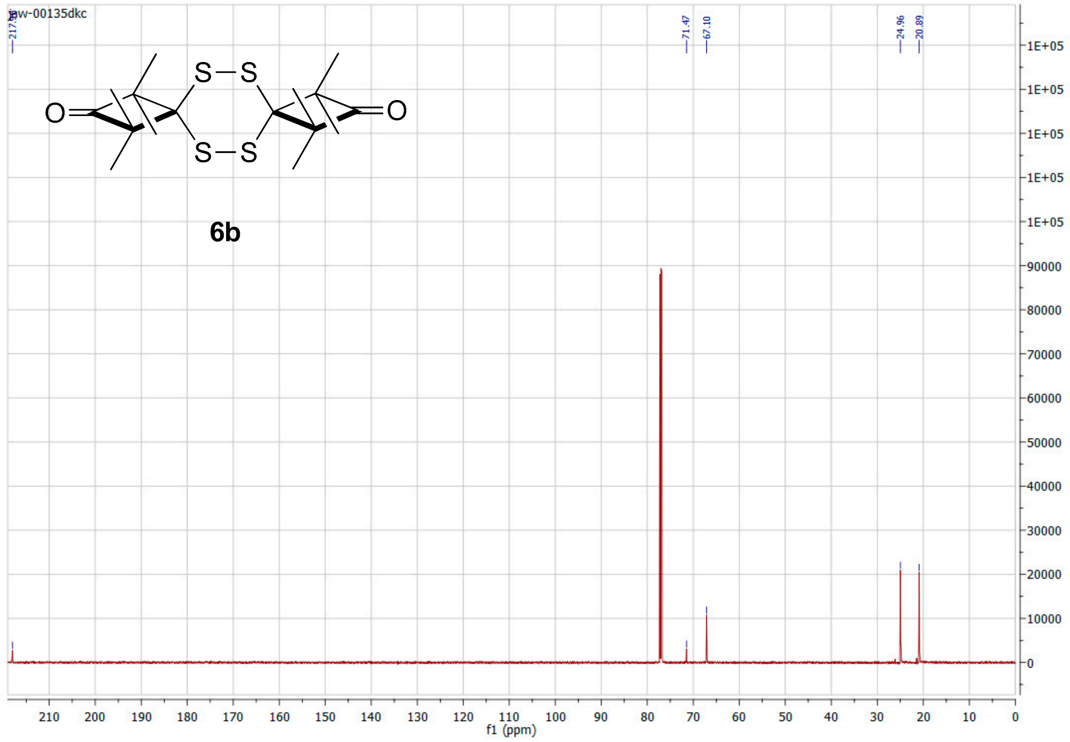

Figure S6. ^{13}C-NMR of 1,1,3,3,8,8,10,10-octamethyl-5,6,11,12-tetrathiadispiro-[3,2,3,3]dodecane-2,9-dione (**6b**) ($CDCl_3$, 151 MHz).

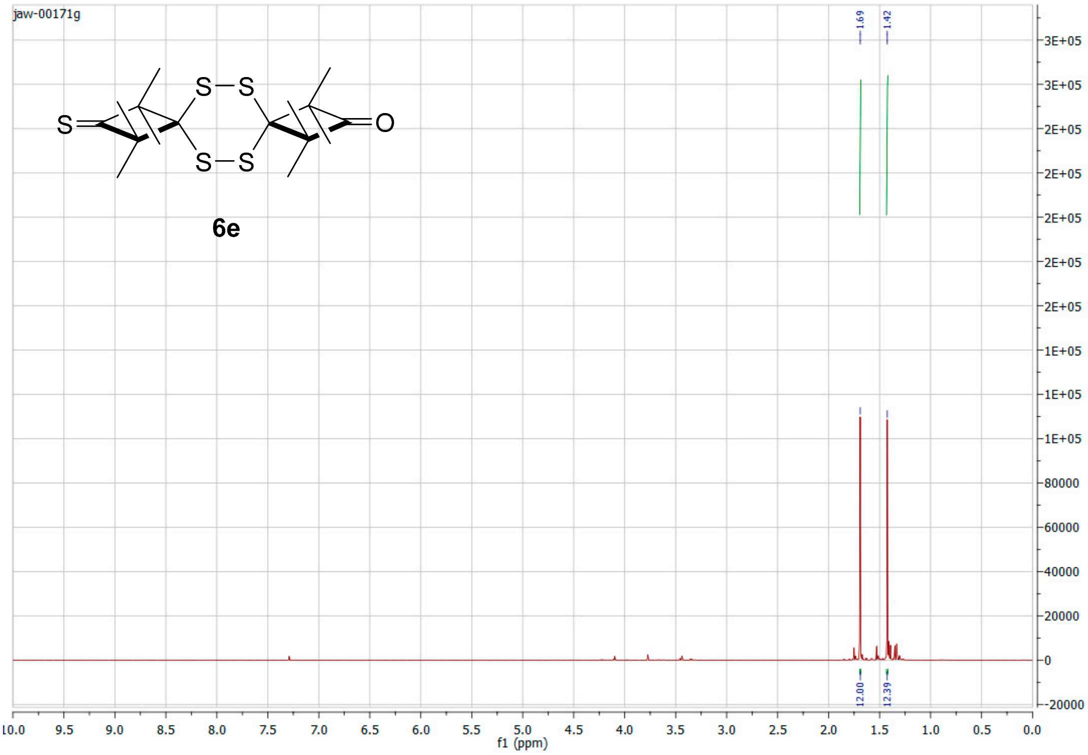

Figure S7. ^{1}H-NMR of 1,1,3,3,8,8,10,10-octamethyl-5,6,11,12-tetrathiadispiro-[3,2,3,3]-9-oxododecane-2-thione (**6e**) ($CDCl_3$, 600 MHz).

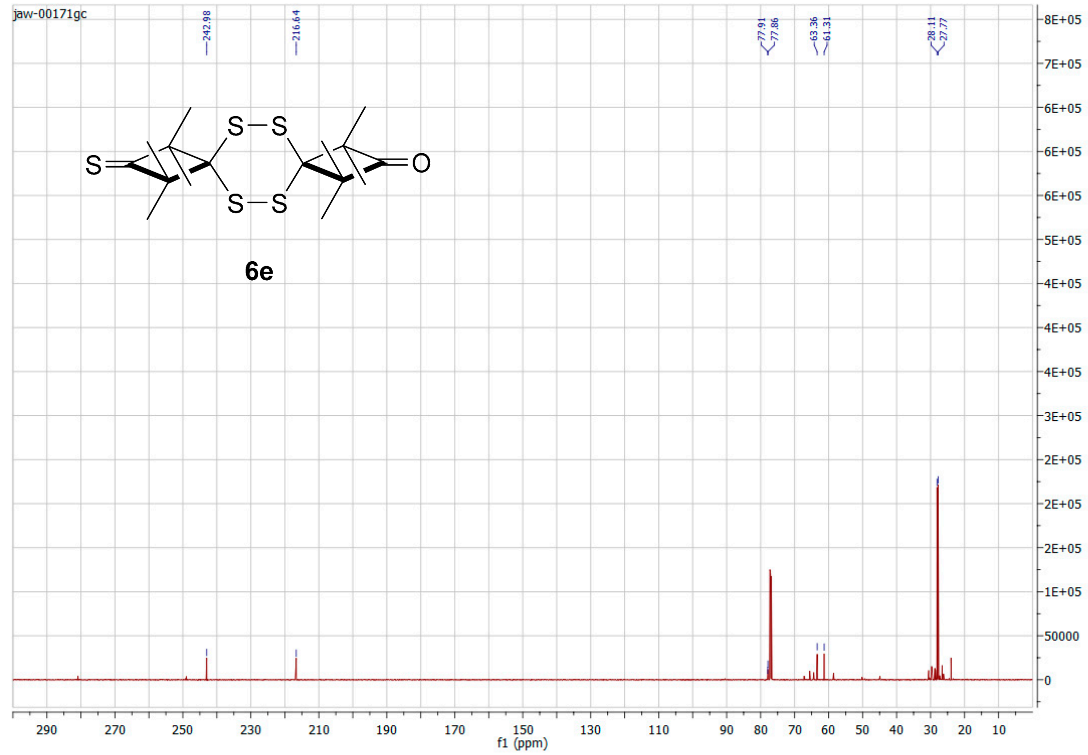

Figure S8. ^{13}C-NMR of 1,1,3,3,8,8,10,10-octamethyl-5,6,11,12-tetrathiadispiro-[3,2,3,3]-9-oxododecane-2-thione (**6e**) ($CDCl_3$, 151 MHz).

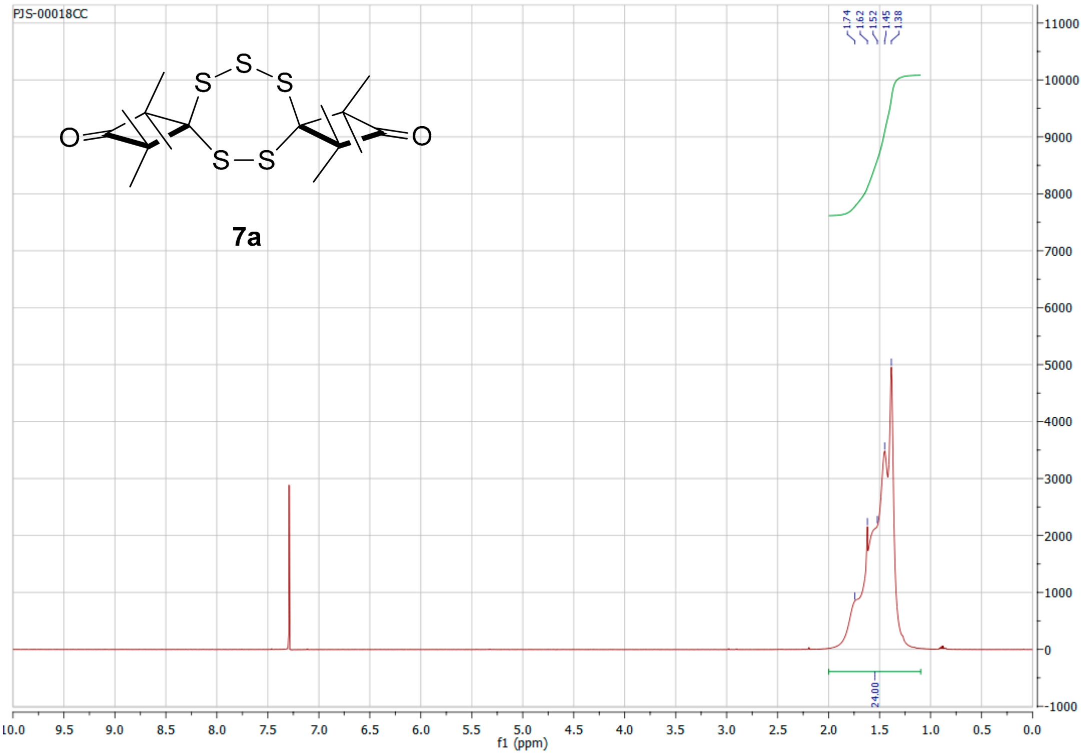

Figure S9. ^{1}H-NMR of 1,1,3,3,8,8,10,10-octamethyl-5,6,11,12,13-pentathiadispiro-[3,2,3,3]tridecane-2,9-dione (**7a**) ($CDCl_3$, 600 MHz)..

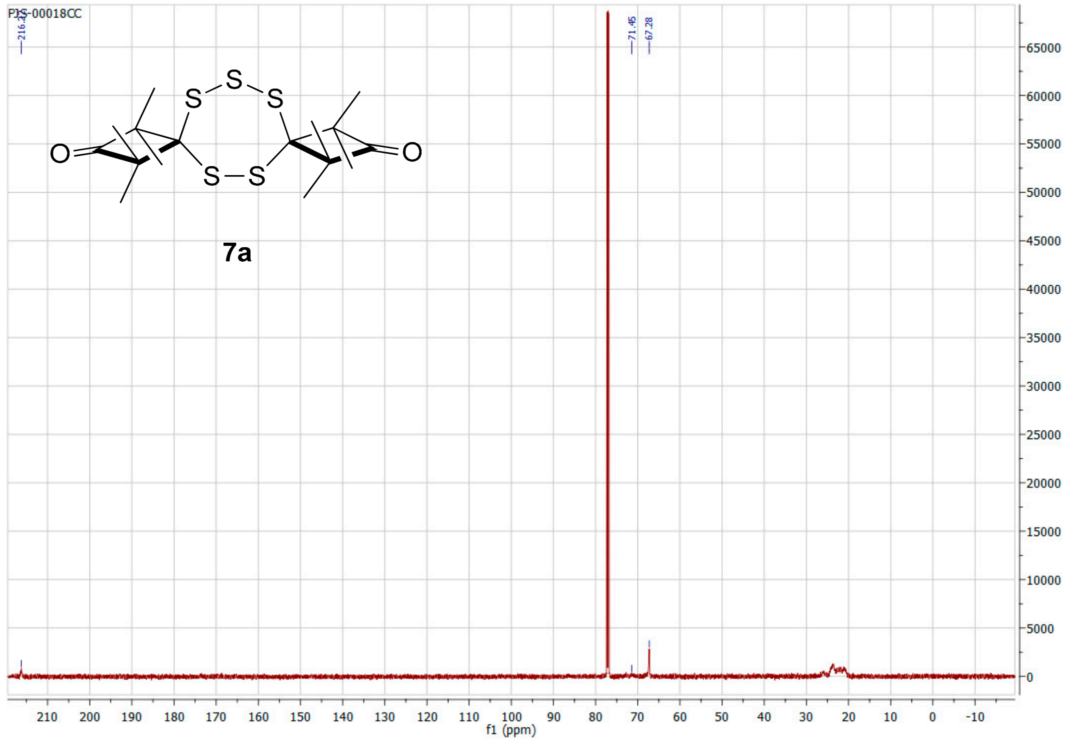

Figure S10. ^{13}C-NMR of 1,1,3,3,8,8,10,10-octamethyl-5,6,11,12,13-pentathiadispiro-[3,2,3,3]tridecane-2,9-dion (**7a**) ($CDCl_3$, 151 MHz).

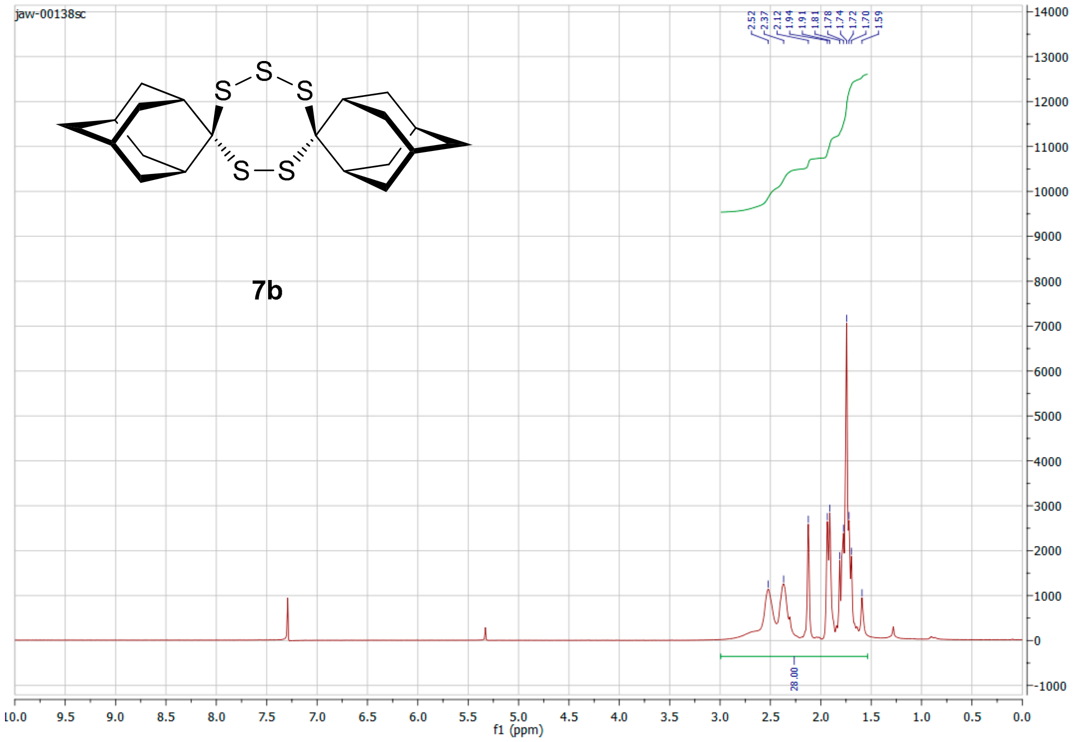

Figure S11. ^{1}H-NMR of dispiro[adamantane-2,4′-(1,2,3,5,6)-pentathiepane-7′,2″-adamantane] (**7b**): ($CDCl_3$, 600 MHz).

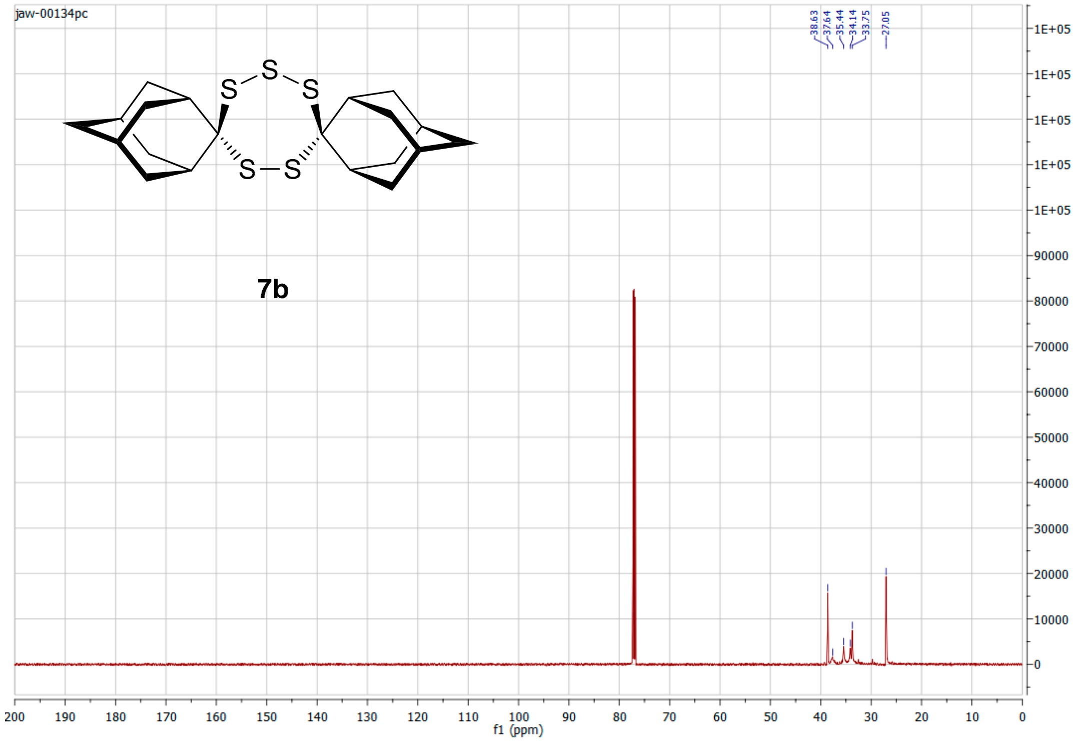

Figure S12. ^{13}C-NMR of dispiro[adamantane-2,4′-(1,2,3,5,6)-pentathiepane-7′,2″-adamantane] (**7b**): ($CDCl_3$, 151 MHz).

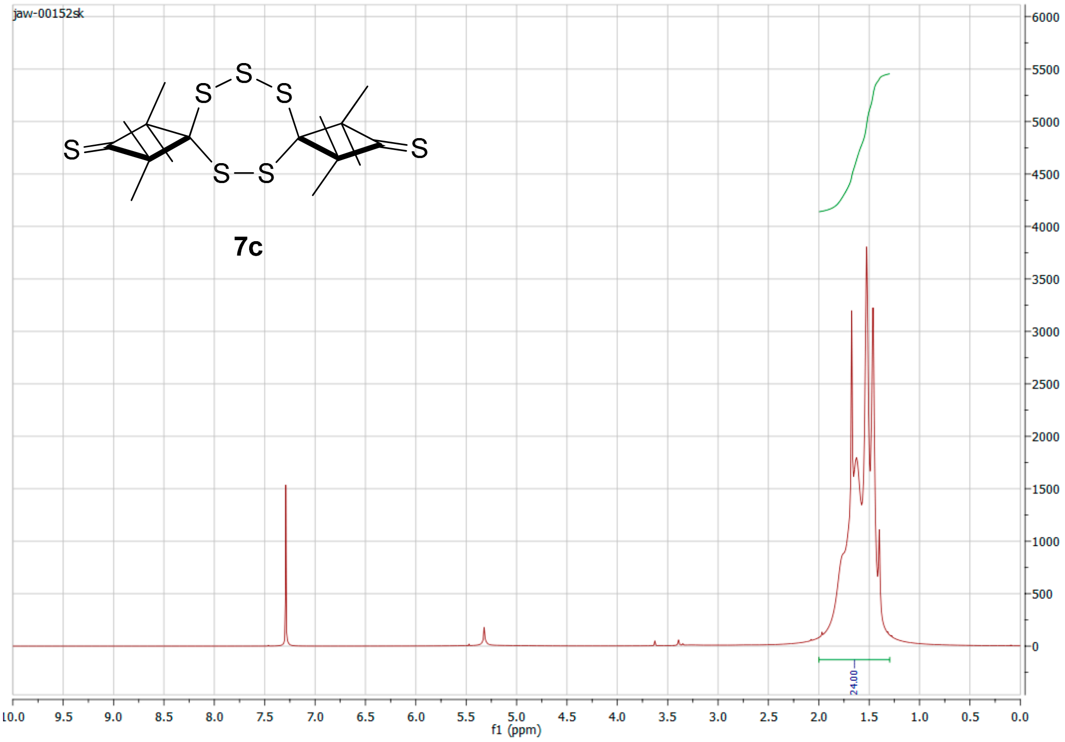

Figure S13. ^{1}H-NMR of 1,1,3,3,8,8,10,10-octamethyl-5,6,11,12,13-pentathiadispiro-[3,2,3,3]tridecane-2,9-dithione (**7c**) ($CDCl_3$, 600 MHz).

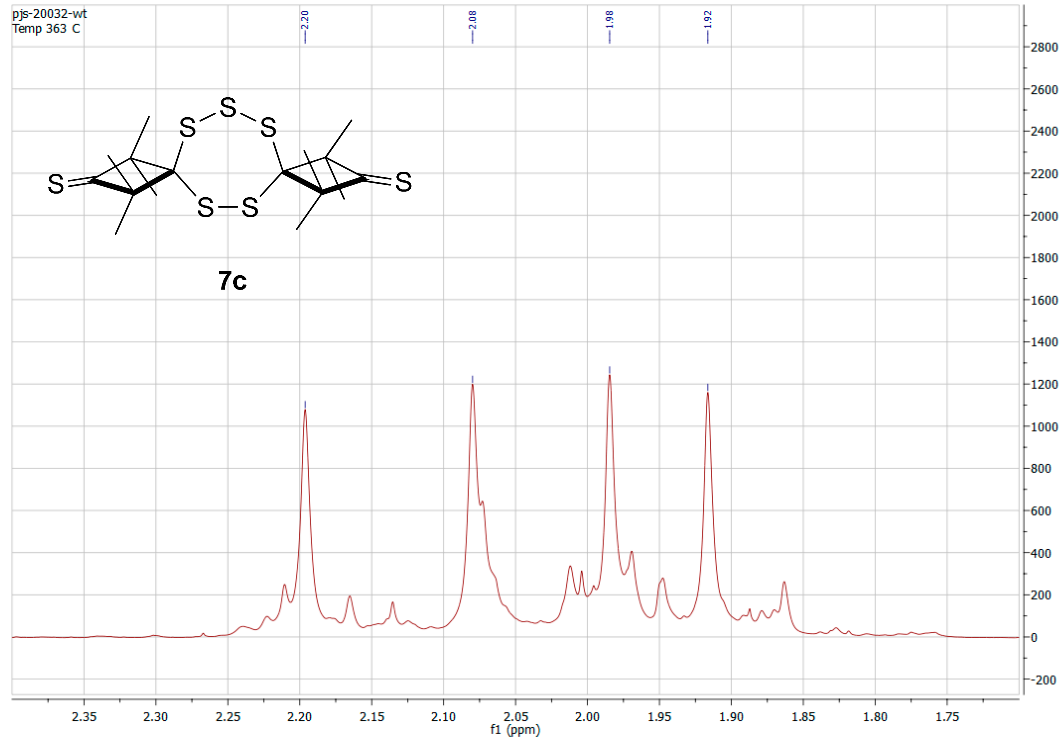

Figure S14. ^{1}H-NMR of 1,1,3,3,8,8,10,10-octamethyl-5,6,11,12,13-pentathiadispiro-[3,2,3,3]tridecane-2,9-dithione (**7c**) (90 °C, C_2Cl_4; D_2O, 600 MHz).

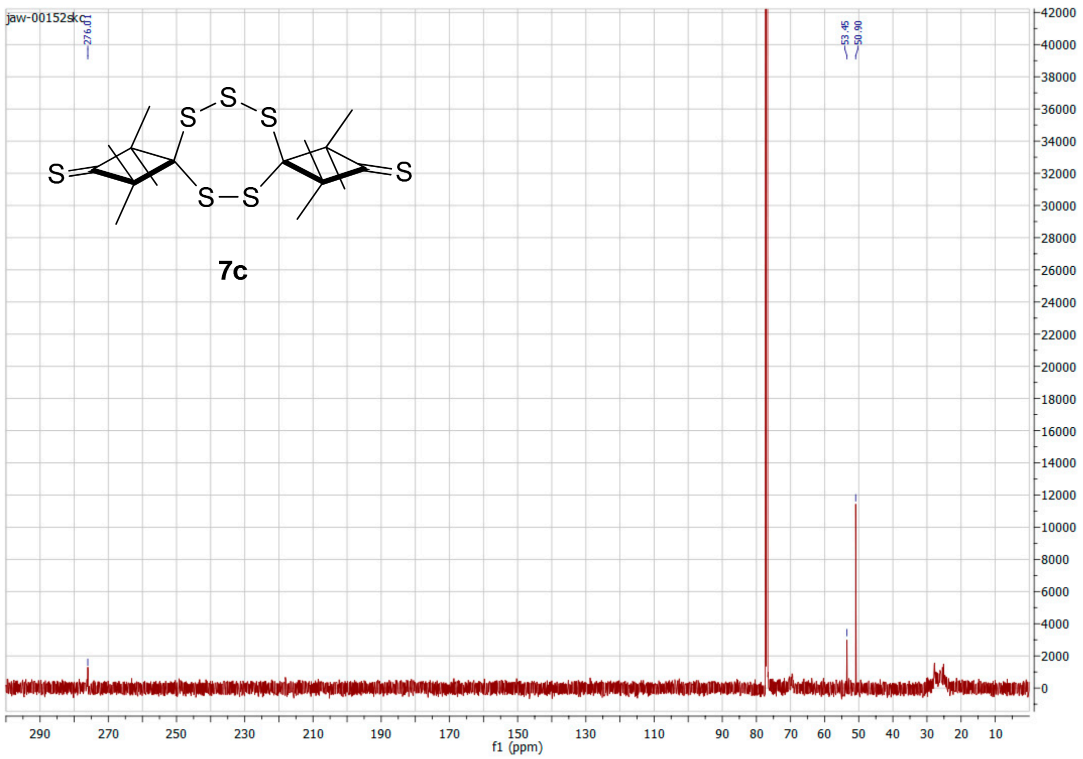

Figure S15. ^{13}C-NMR of 1,1,3,3,8,8,10,10-octamethyl-5,6,11,12,13-pentathiadispiro-[3,2,3,3]tridecane-2,9-dithione (**7c**) ($CDCl_3$, 151 MHz).

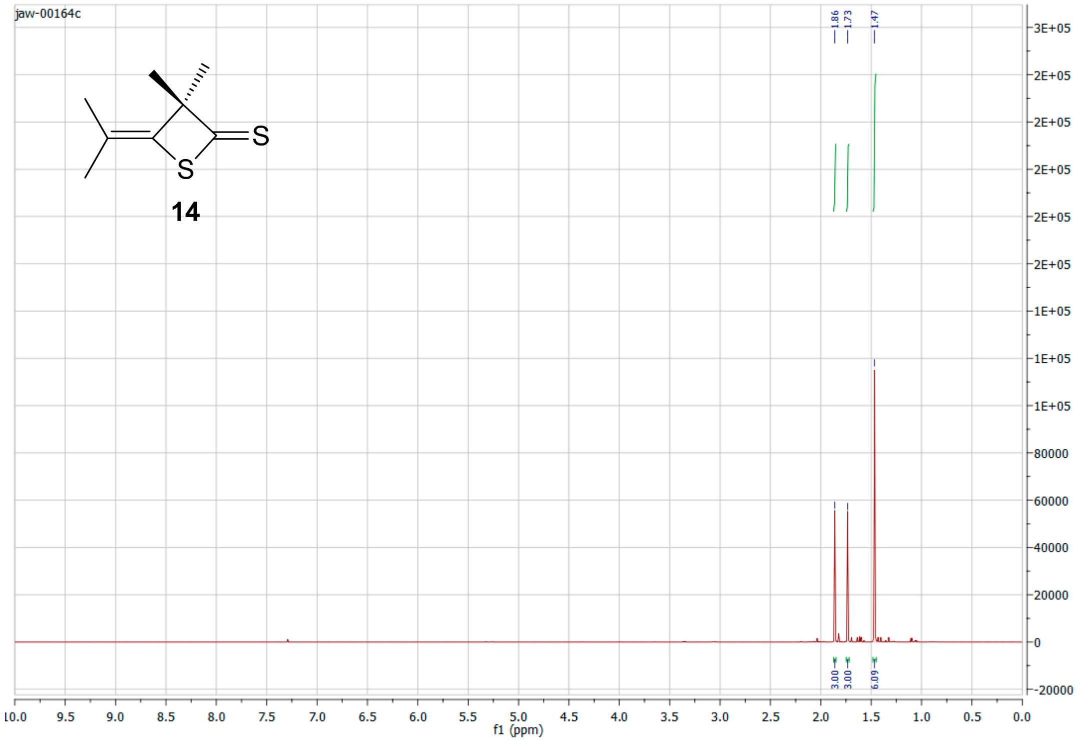

Figure S16. ^{1}H-NMR of a 3,3-dimethyl-4-(propan-2-ylidene)thietane-2-thione (**14**) ($CDCl_3$, 600 MHz).

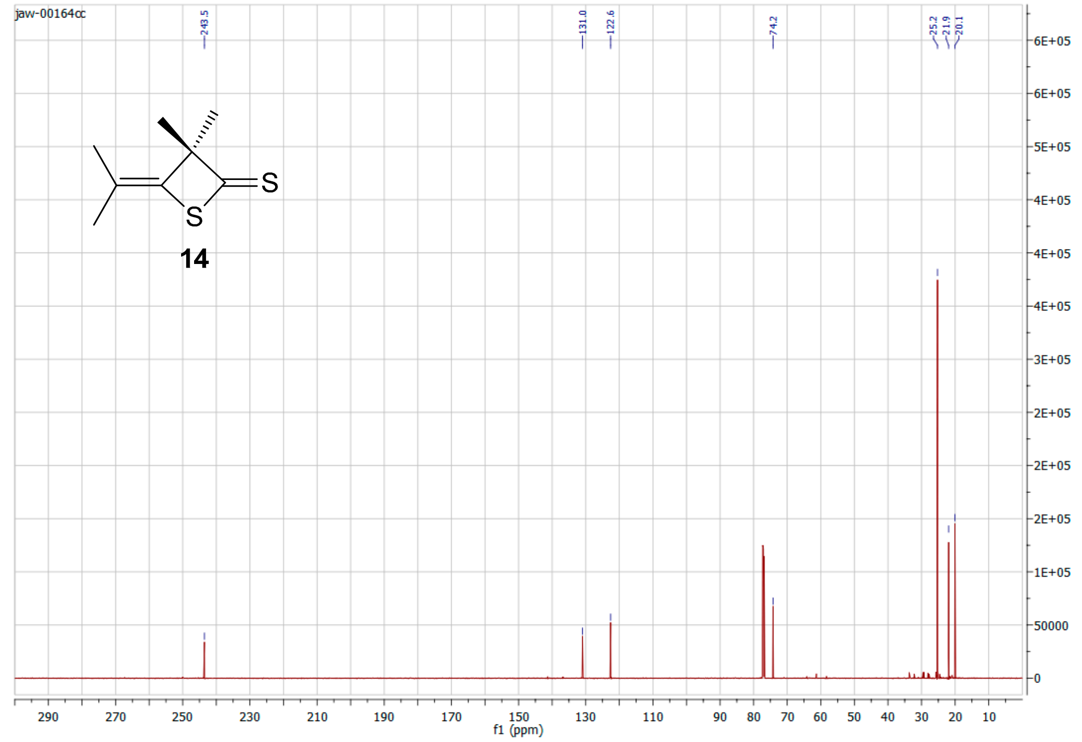

Figure S17. ^{13}C-NMR of a 3,3-dimethyl-4-(propan-2-ylidene)thietane-2-thione (**14**) ($CDCl_3$, 151 MHz).

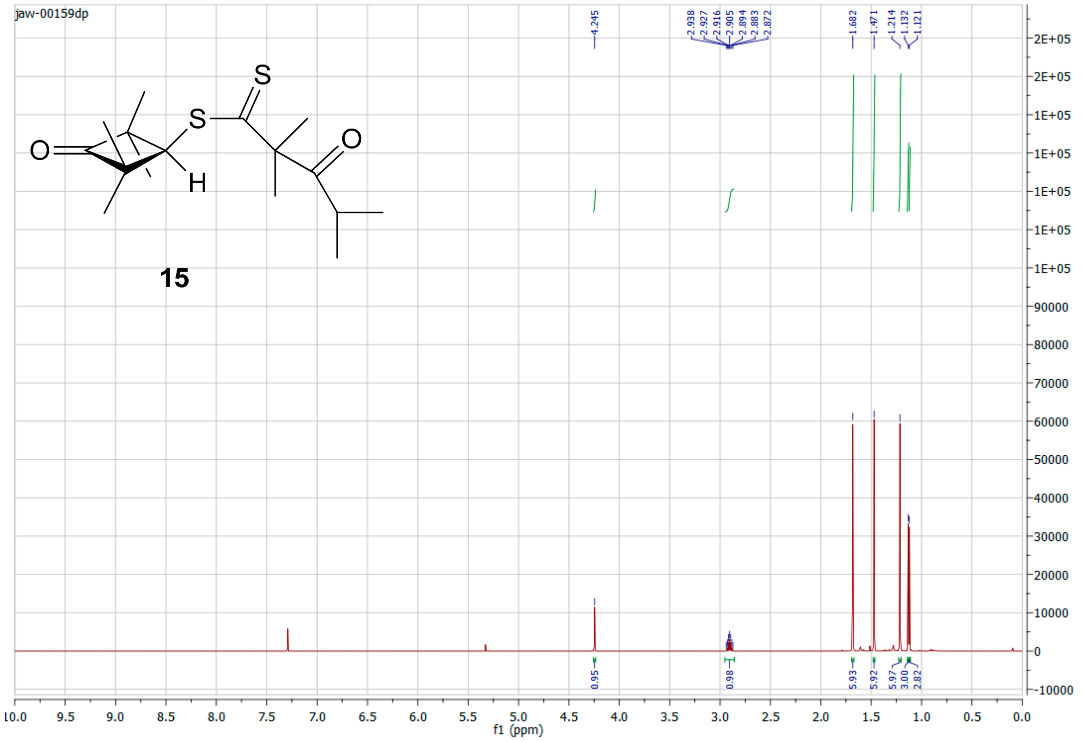

Figure S18. ^{1}H-NMR of 2,2,4,4-tetramethyl-3-oxocyclobutyl-2′,2′,4′-Trimethyl-3′-oxopentanedithioate (**15**) ($CDCl_3$, 600 MHz).

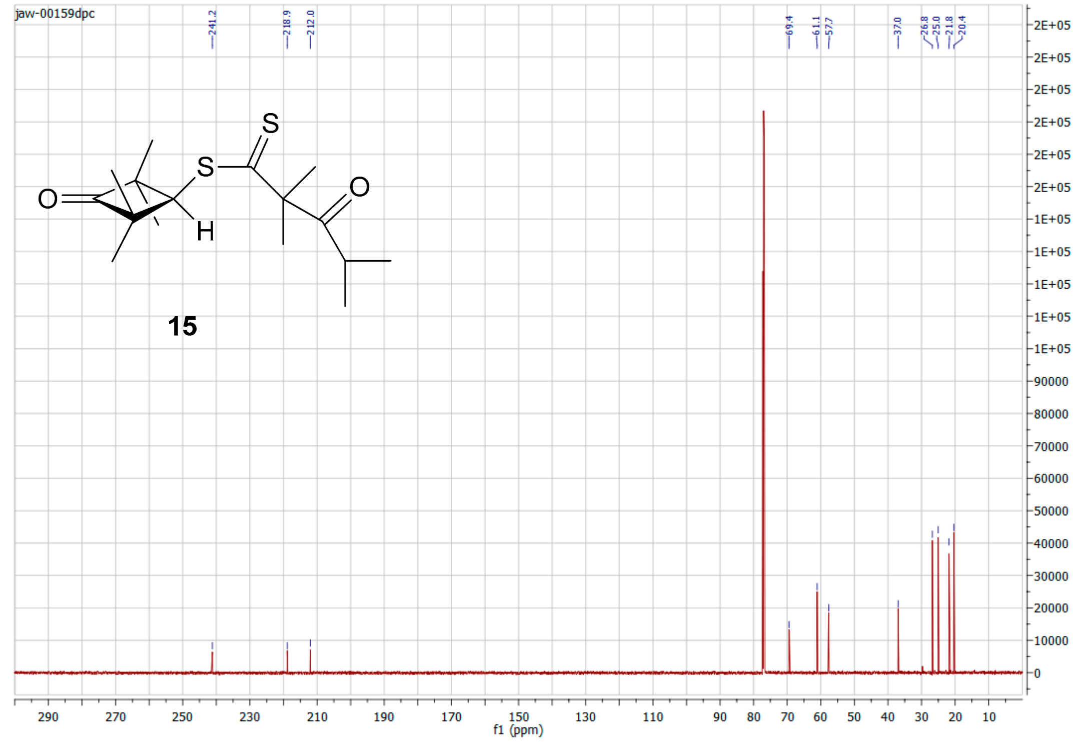

Figure S19. ^{13}C-NMR of 2,2,4,4-tetramethyl-3-oxocyclobutyl-2′,2′,4′-trimethyl-3′-oxopentanedithioate (**15**) ($CDCl_3$, 151 MHz).

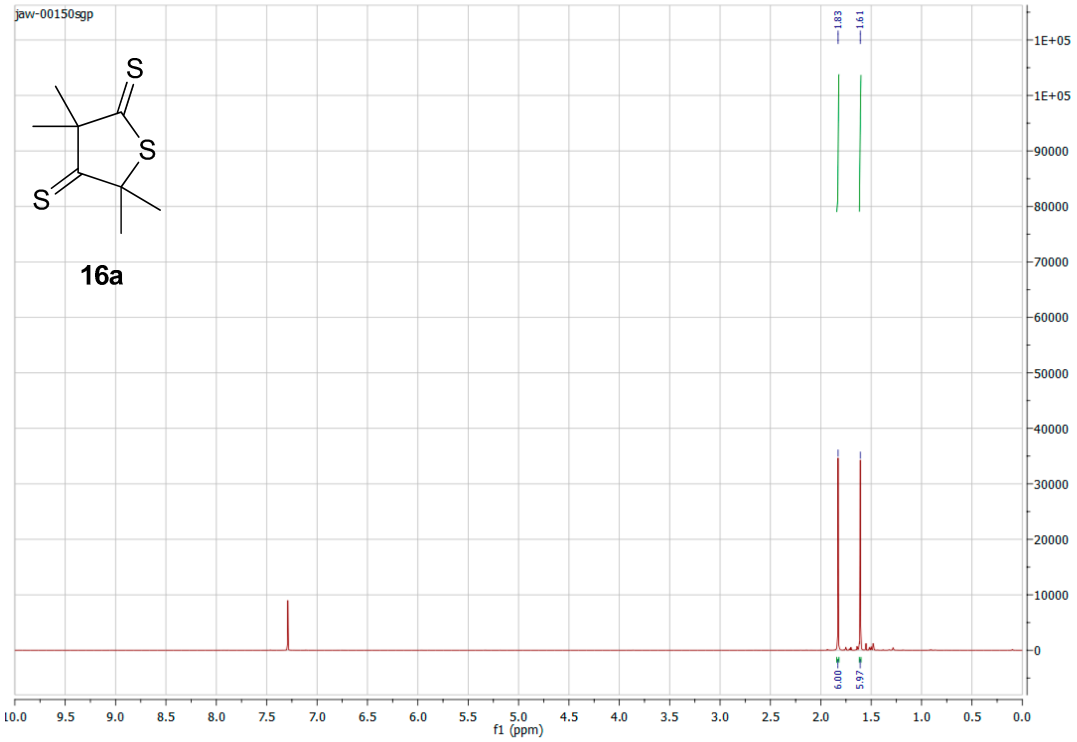

Figure S20. ^{1}H-NMR of 3,3,5,5-tetramethyl-4-thioxothiolane-2-thione (**16a**) ($CDCl_3$, 600 MHz).

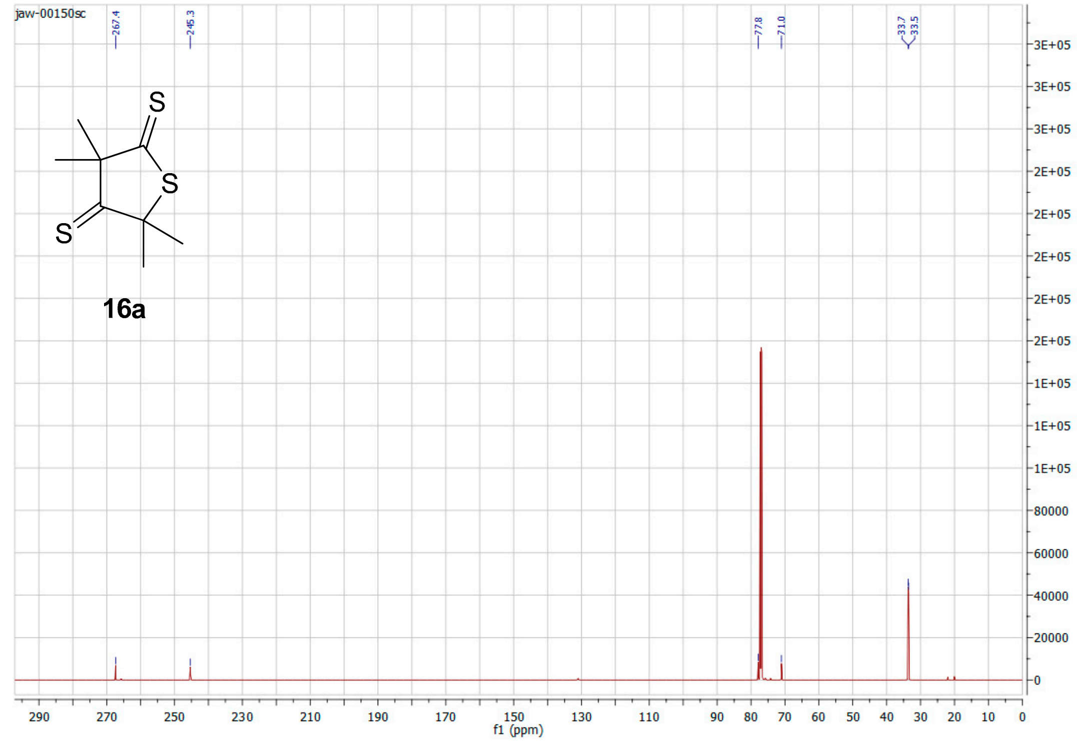

Figure S21. ^{13}C-NMR of 3,3,5,5-tetramethyl-4-thioxothiolane-2-thione (**16a**) ($CDCl_3$, 151 MHz).

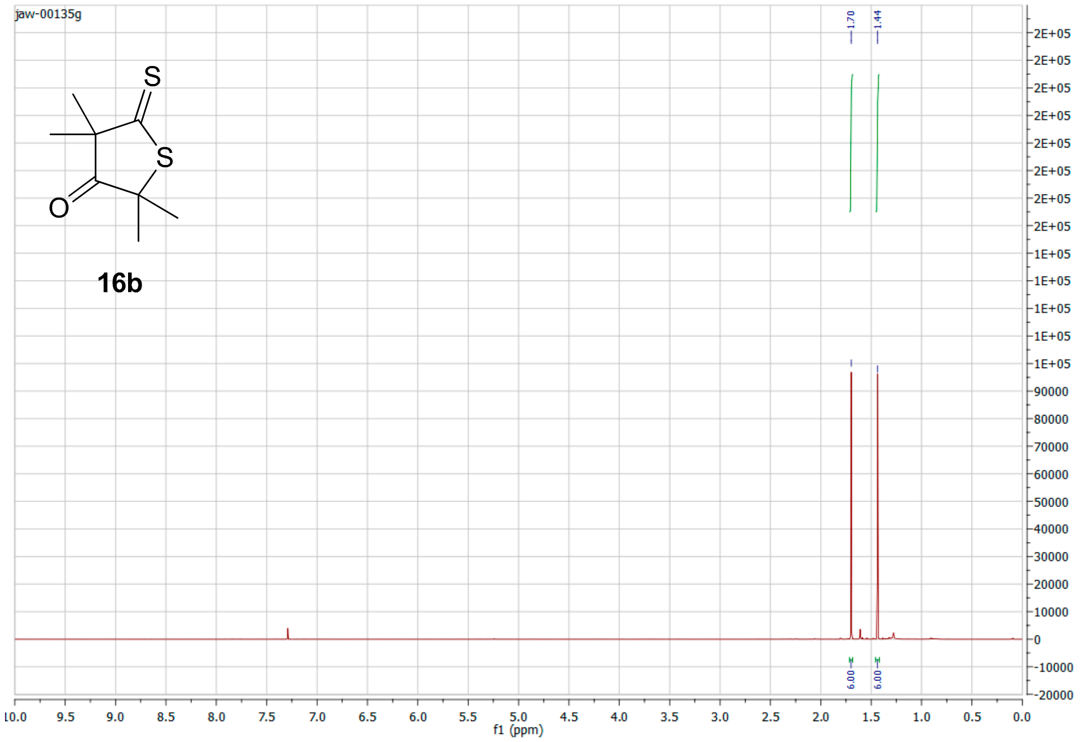

Figure S22. ^{1}H-NMR of 3,3,5,5-tetramethyl-2-thioxothiolane-4-one (**16b**) ($CDCl_3$, 600 MHz).

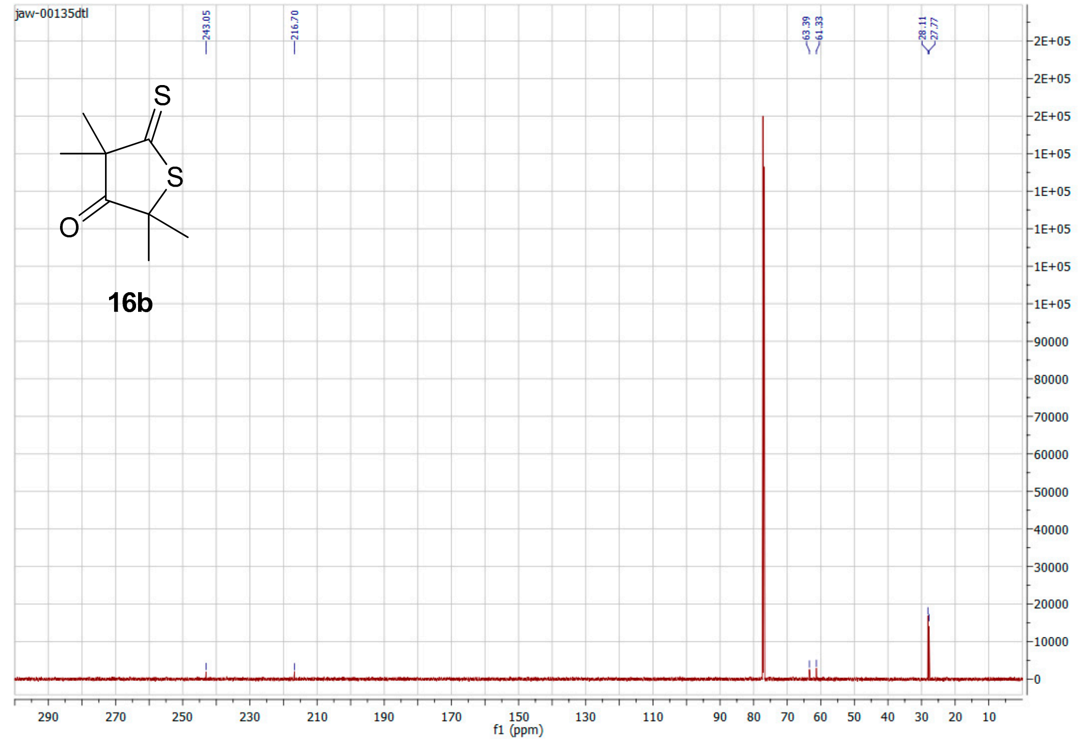

Figure S23. ^{13}C-NMR of 3,3,5,5-tetramethyl-2-thioxothiolane-4-one (**16b**) ($CDCl_3$, 151 MHz).

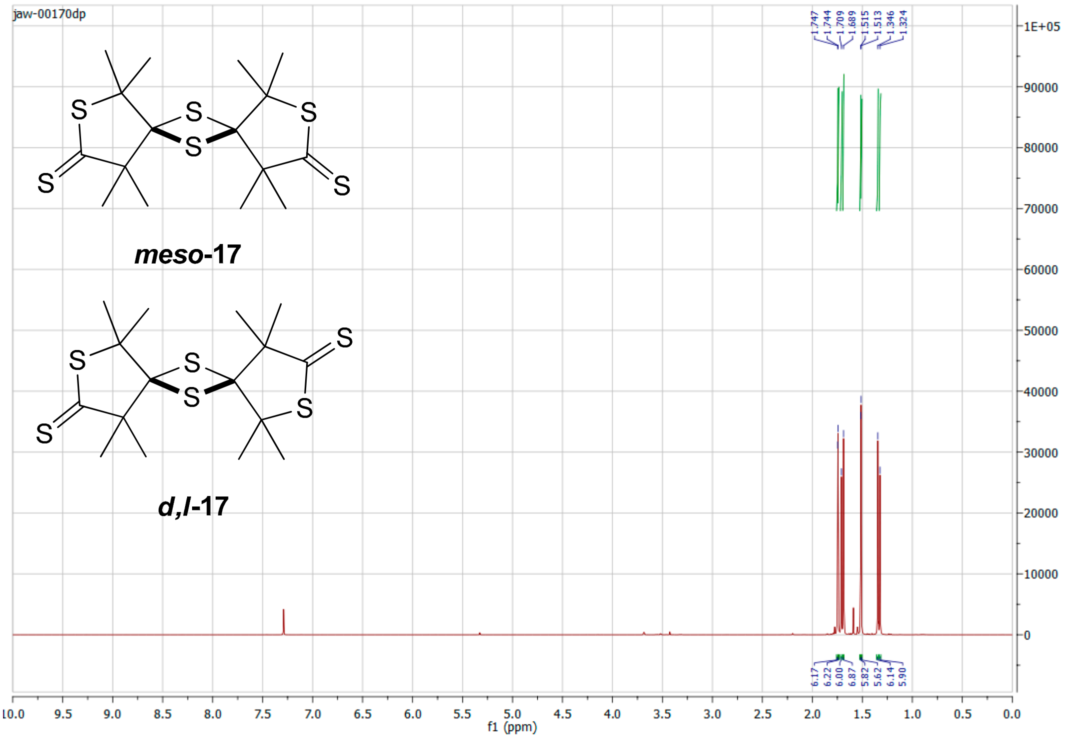

Figure S24. ¹H-NMR of a mixture of 1,1,4,4,8,8,11,11-octamethyl-2,6,9,12-tetrathiadispiro[4.1.47.15]dodecane-3,10-dithione (***meso*-17**) and 1,1,4,4,8,8,11,11-octamethyl-2,6,10,12-tetrathiadispiro[4.1.47.15]dodecane-3,9-dithione (***d,l*-17**) ($CDCl_3$, 600 MHz).

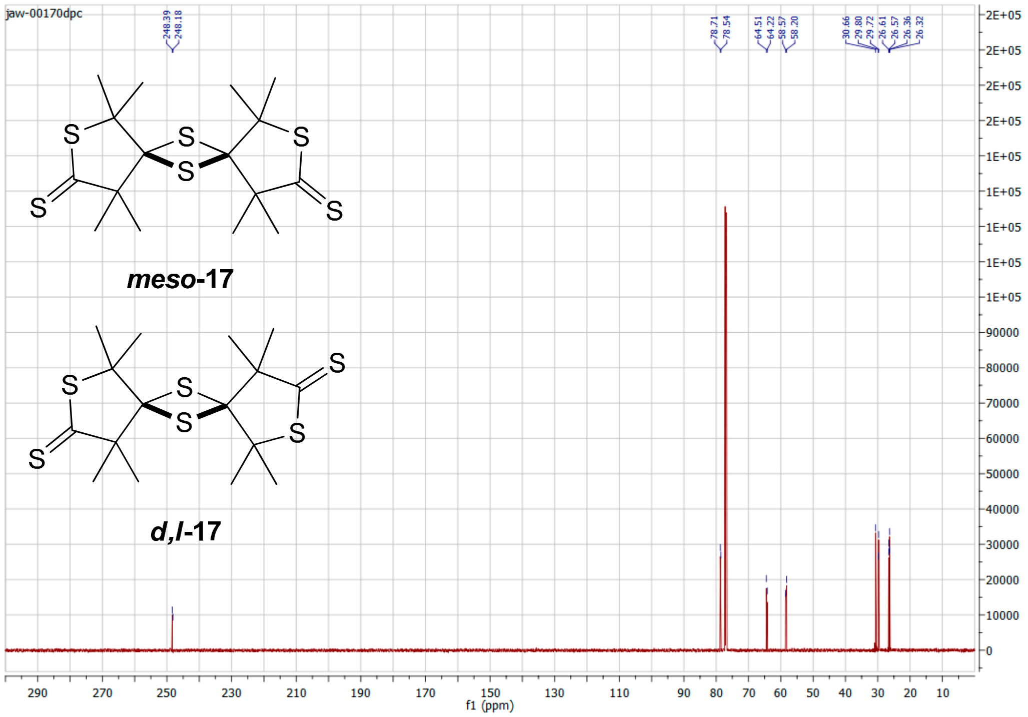

Figure S25. ¹³C-NMR of a mixture of 1,1,4,4,8,8,11,11-octamethyl-2,6,9,12-tetrathiadispiro[4.1.47.15]dodecane-3,10-dithione (***meso*-17**) and 1,1,4,4,8,8,11,11-octamethyl-2,6,10,12-tetrathiadispiro[4.1.47.15]dodecane-3,9-dithione (***d,l*-17**) ($CDCl_3$, 151 MHz).

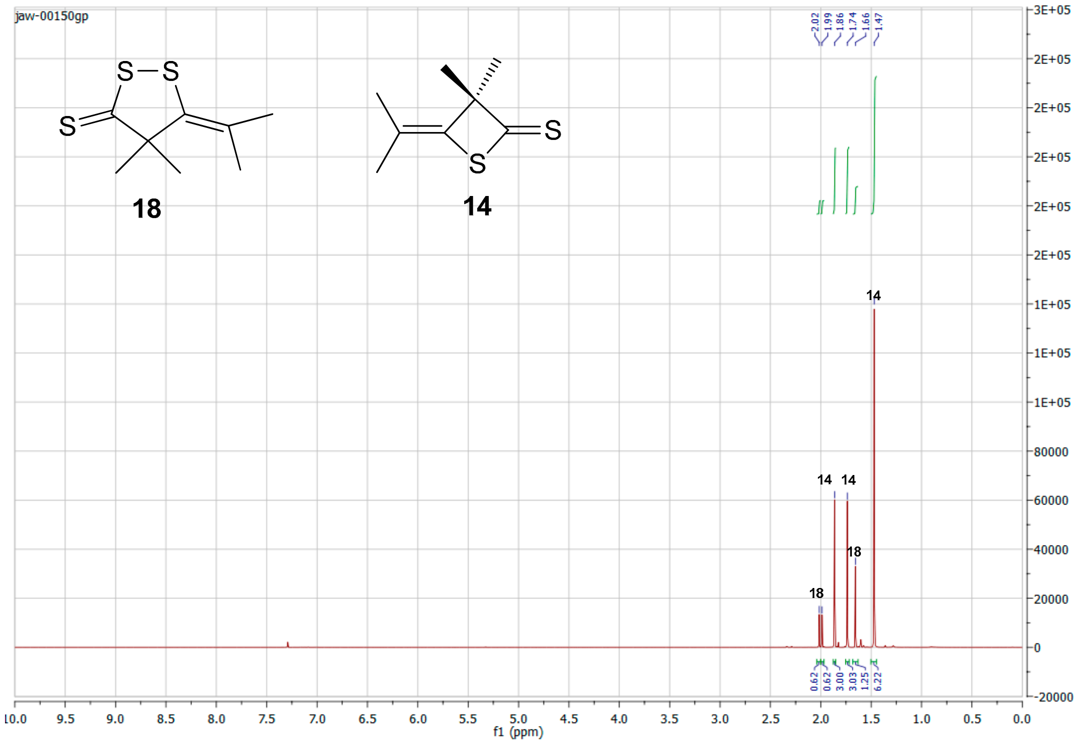

Figure S26. ^{1}H-NMR of 4,4-dimethyl-5-(propan-2-ylidene)-1,2-dithiolane-3-thione (**18**) in a mixture with 3,3-dimethyl-4-(propan-2-ylidene)thietane-2-thione (**14**) ($CDCl_3$, 600 MHz).

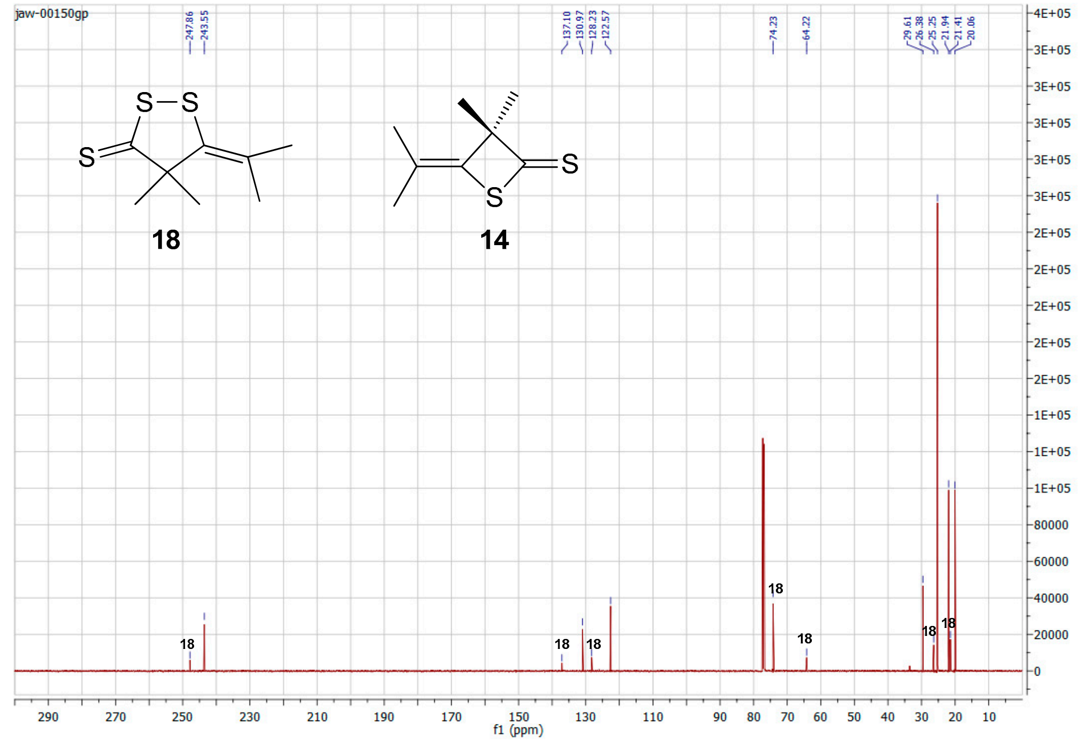

Figure S27. ^{13}C-NMR of 4,4-dimethyl-5-(propan-2-ylidene)-1,2-dithiolane-3-thione (**18**) in a mixture with 3,3-dimethyl-4-(propan-2-ylidene)thietane-2-thione (**14**) ($CDCl_3$, 151 MHz).

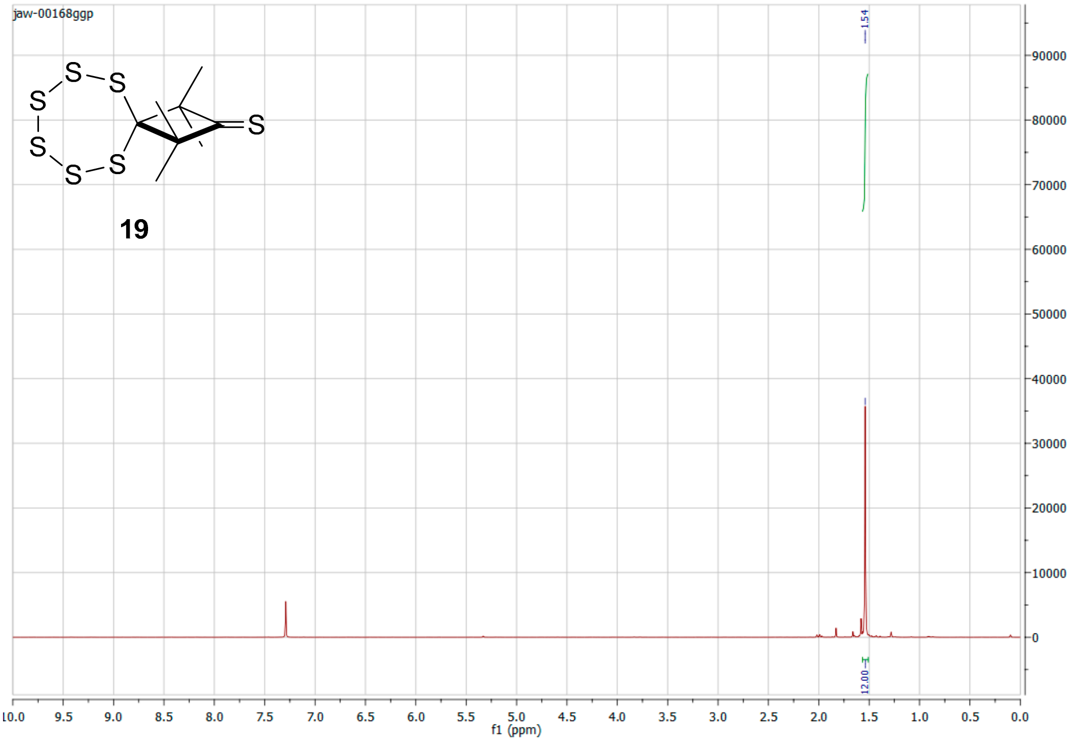

Figure S28. ^{1}H-NMR of 1,1,3,3-tetramethyl-5,6,7,8,9,10-hexathiaspiro[3.6]decane-2-thione (**19**): ($CDCl_3$, 600 MHz).

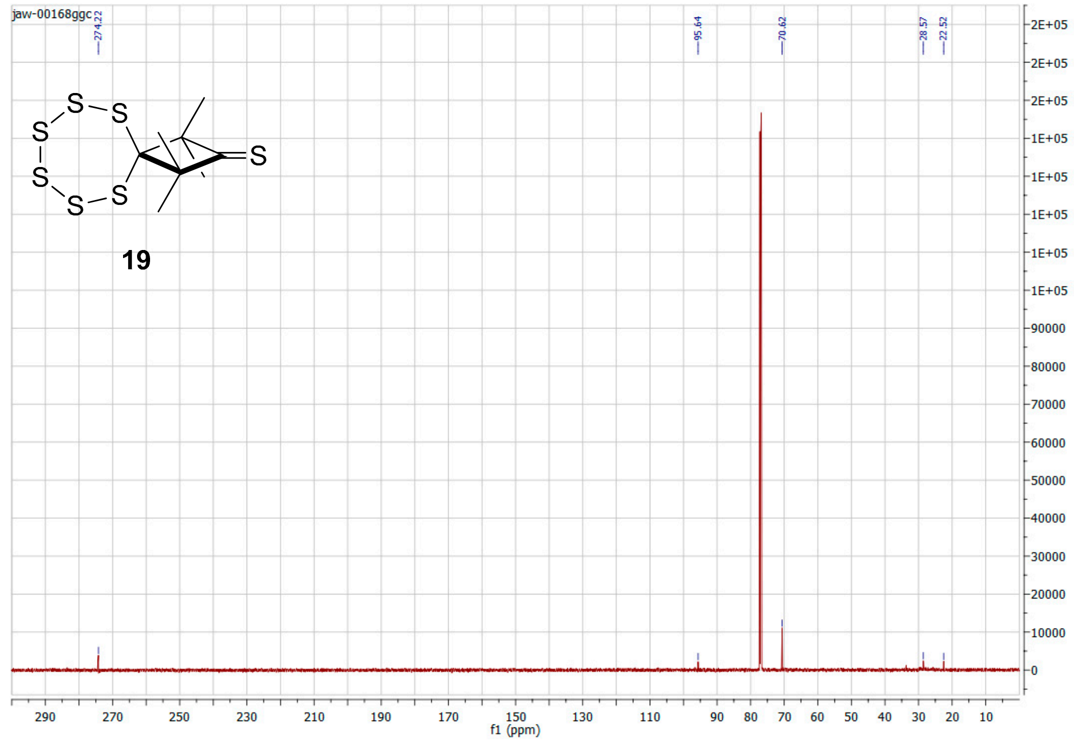

Figure S29. ^{13}C-NMR of 1,1,3,3-tetramethyl-5,6,7,8,9,10-hexathiaspiro[3.6]decane-2-thione (**19**): ($CDCl_3$, 151 MHz).

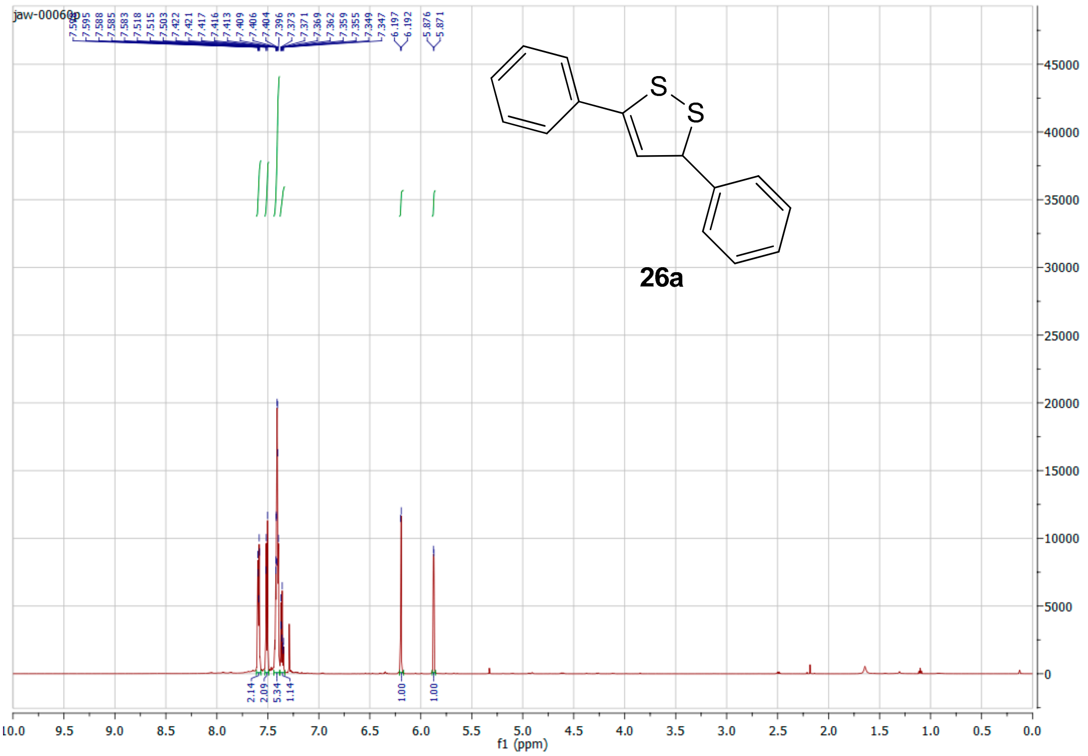

Figure S30. ^{1}H-NMR of 3,5-diphenyl-3*H*-1,2-dithiole (**26a**): ($CDCl_3$, 600 MHz).

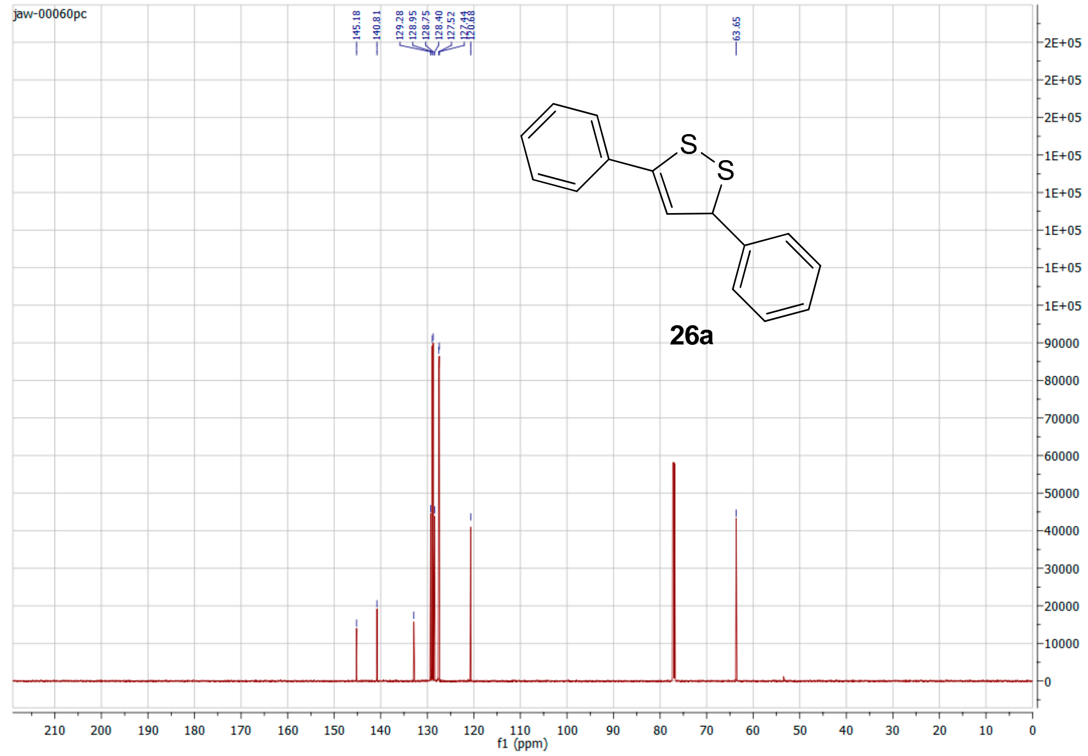

Figure S31. ^{13}C-NMR of 3,5-diphenyl-3*H*-1,2-dithiole (**26a**): ($CDCl_3$, 151 MHz).

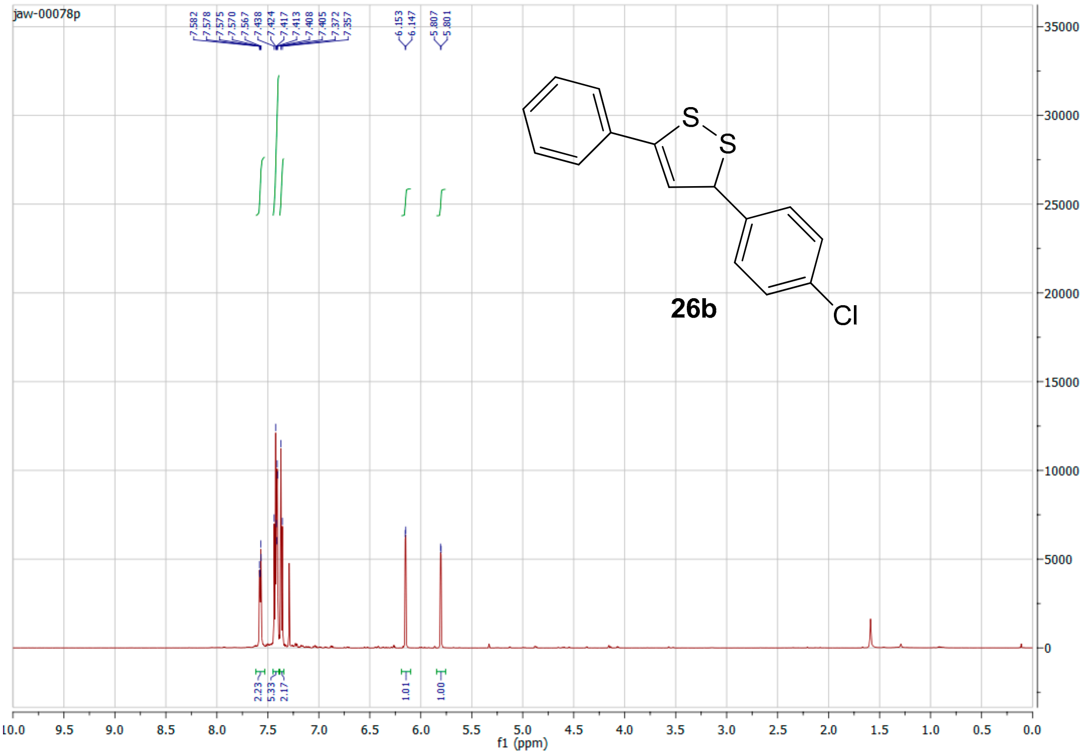

Figure S32. ^{1}H-NMR of 3-(4-chlorophenyl)-5-phenyl-3*H*-1,2-dithiole (**26b**): ($CDCl_3$, 600 MHz).

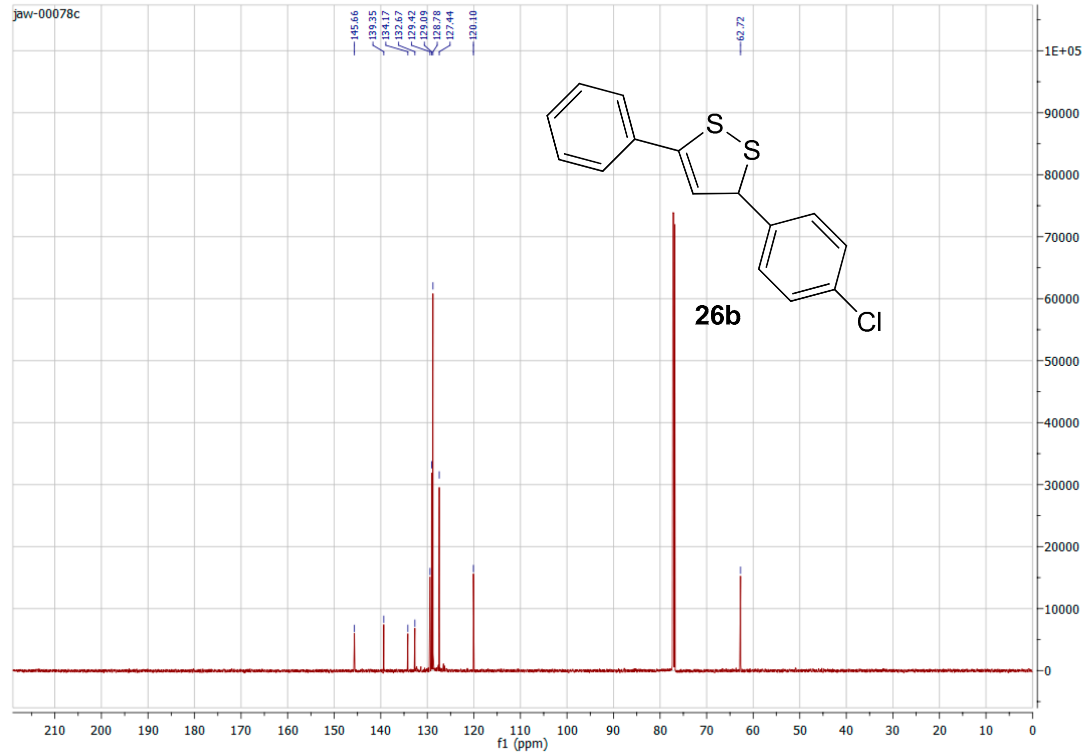

Figure S33. ^{13}C-NMR of 3-(4-chlorophenyl)-5-phenyl-3*H*-1,2-dithiole (**26b**): ($CDCl_3$, 151 MHz).

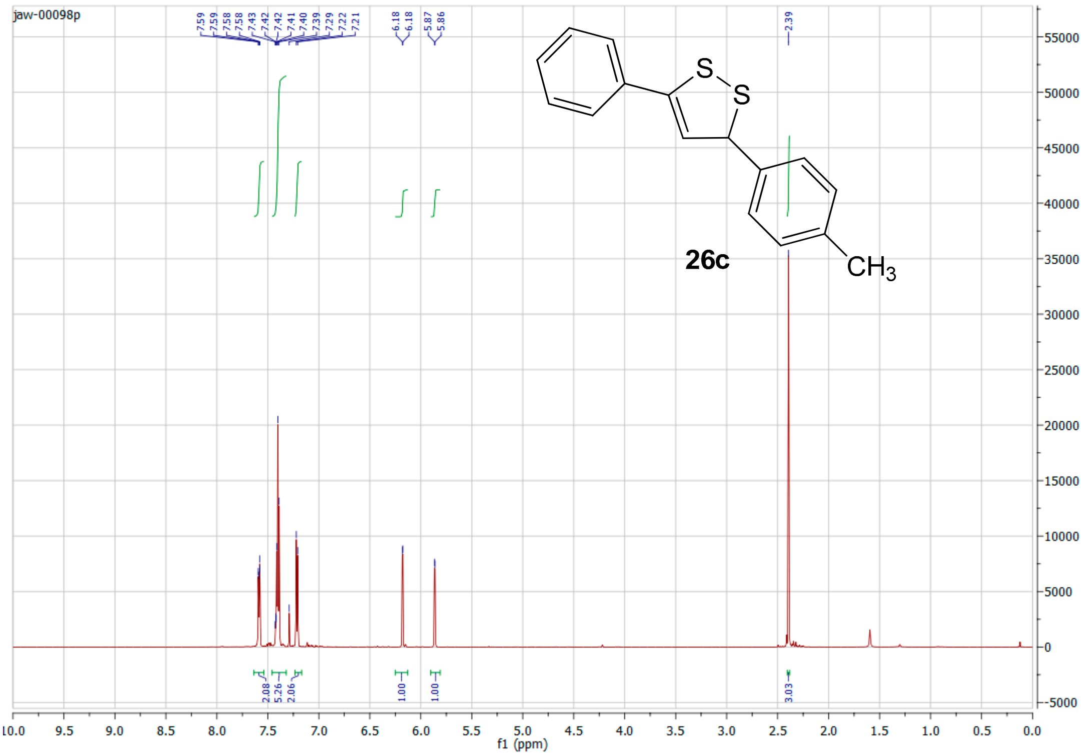

Figure S34. ^{1}H-NMR of 3-(4-methylphenyl)-5-phenyl-3*H*-1,2-dithiole (**26c**): ($CDCl_3$, 600 MHz).

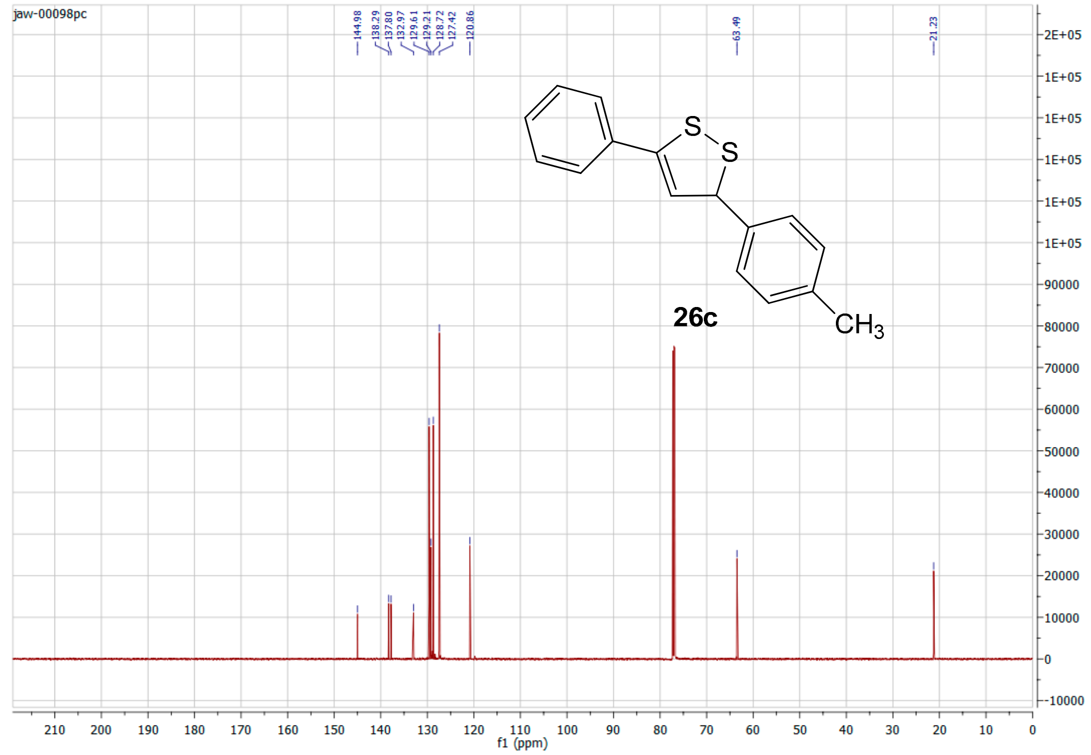

Figure S35. ^{13}C-NMR of 3-(4-methylphenyl)-5-phenyl-3*H*-1,2-dithiole (**26c**): ($CDCl_3$, 151 MHz).

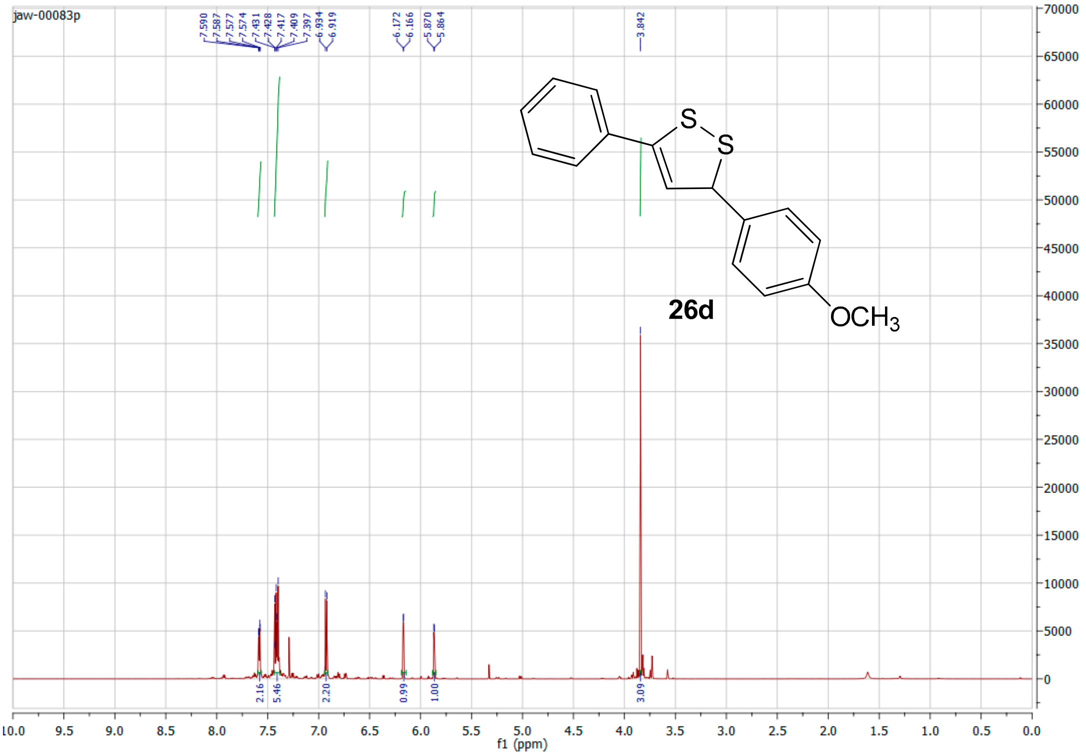

Figure S36. ^{1}H-NMR of 3-(4-methoxyphenyl)-5-phenyl-3*H*-1,2-dithiole (**26d**): ($CDCl_3$, 600 MHz).

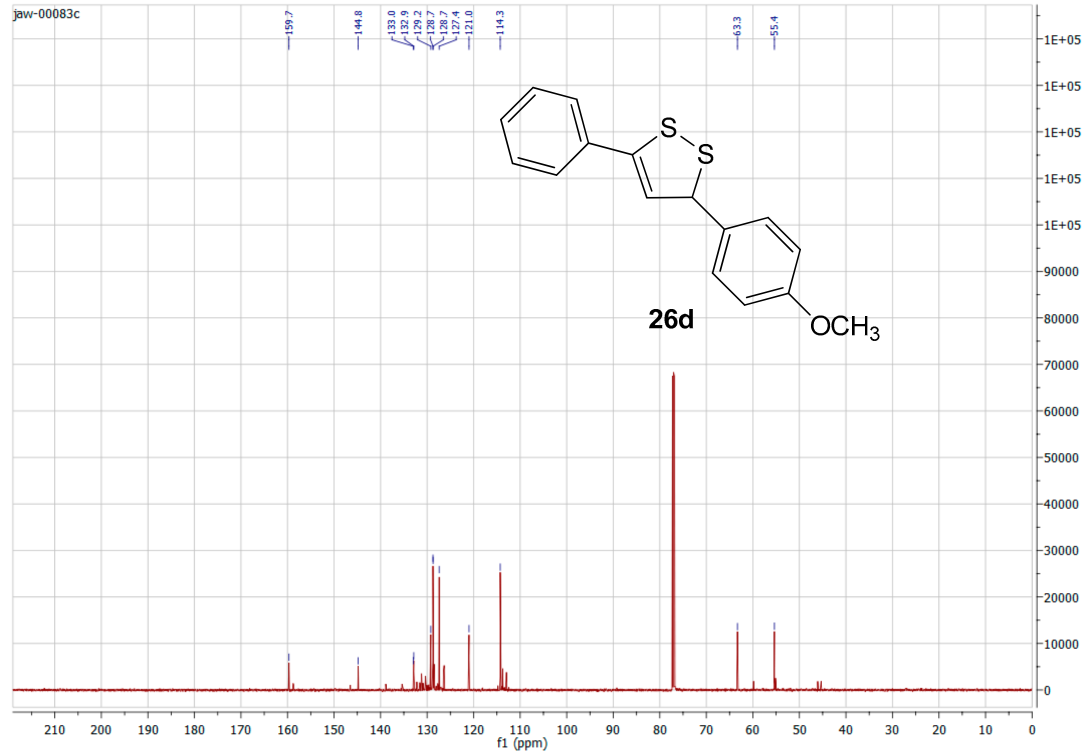

Figure S37. ^{13}C-NMR of 3-(4-methoxyphenyl)-5-phenyl-3*H*-1,2-dithiole (**26d**): ($CDCl_3$, 151 MHz).

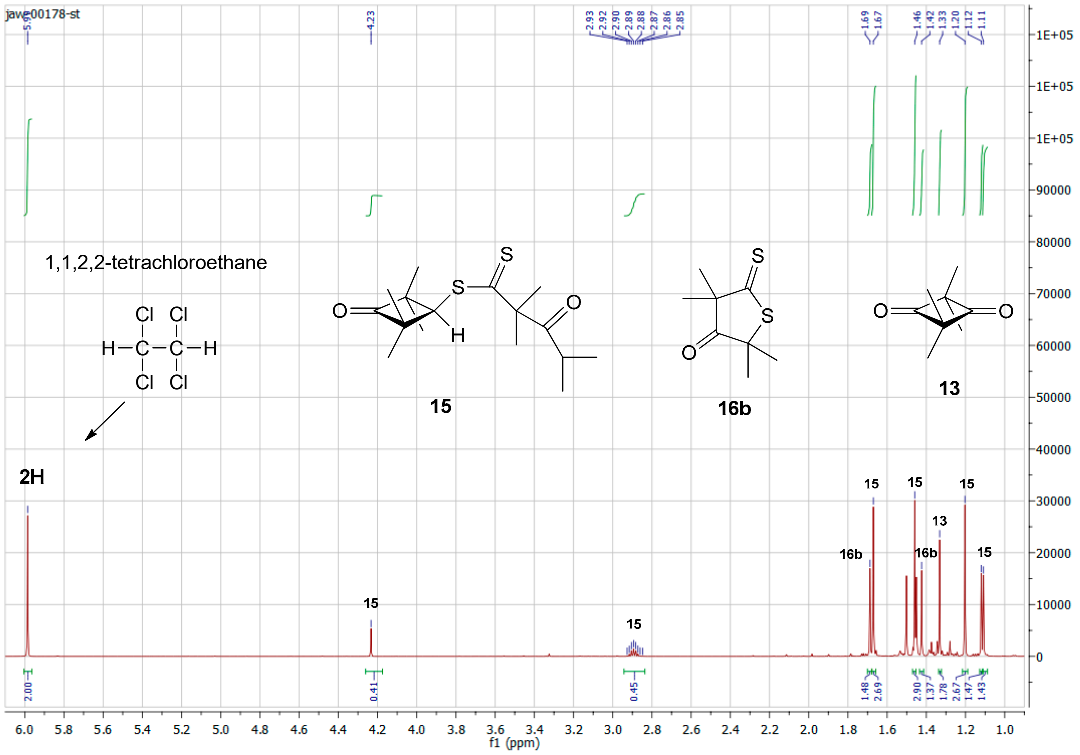

Figure S38. ^{1}H-NMR of crude mixture after conversion of thioketones **2b** in the presence of fluoride anion and absence of S_8 (Procedure II) ($CDCl_3$, 600 MHz).

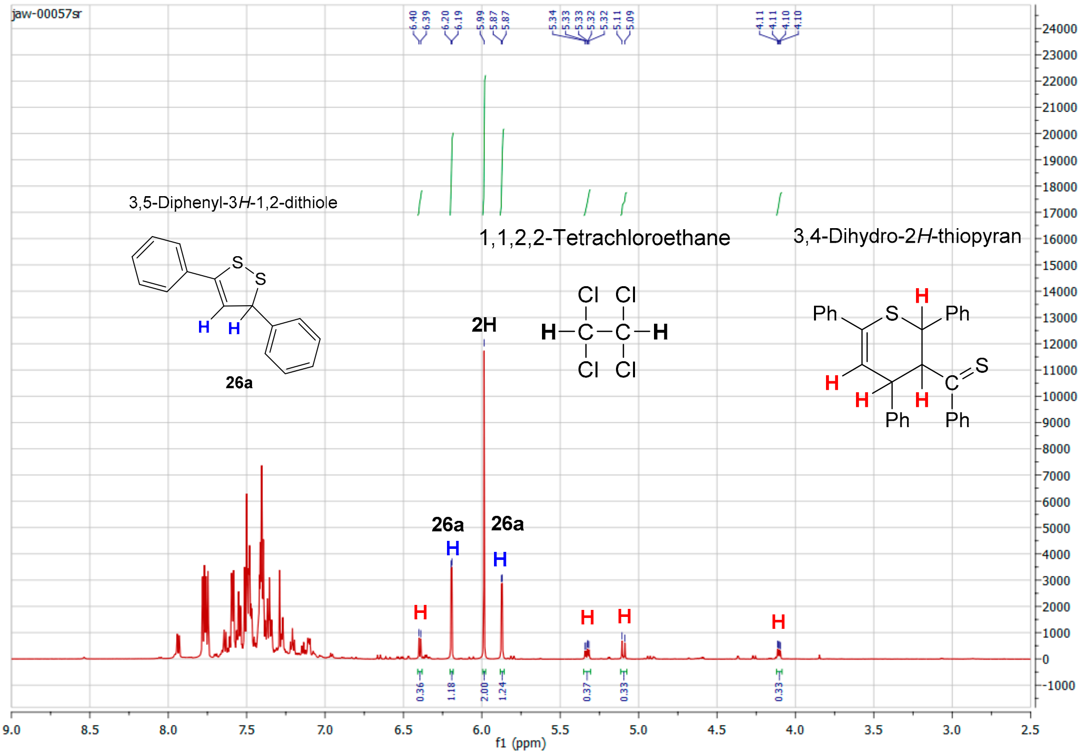

Figure S39. ^{1}H-NMR of crude mixture after sulfurization of thiochalcone **3a** (Procedure I) ($CDCl_3$, 600 MHz).
